# Systematic review and meta-analysis of the safety of chloroquine and hydroxychloroquine from randomized controlled trials on malarial and non-malarial conditions

**DOI:** 10.1186/s13643-021-01835-x

**Published:** 2021-11-04

**Authors:** Mayra Souza Botelho, Fernanda Bolfi, Renata Giacomini Occhiuto Ferreira Leite, Mauro Salles Ferreira Leite, Luisa Rocco Banzato, Luiza Teixeira Soares, Thaina Oliveira Felicio Olivatti, Amanda Sampaio Mangolim, Flávia Ramos Kazan Oliveira, Luciana Patrícia Fernandes Abbade, Joelcio Francisco Abbade, Ricardo Augusto Monteiro de Barros Almeida, Julia Simões Corrêa Galendi, Lehana Thabane, Vania dos Santos Nunes-Nogueira

**Affiliations:** 1 Department of Internal Medicine, São Paulo State University/UNESP, Medical School, Sao Paulo, Brazil; 2 Department of Infectious Diseases, Dermatology, Imaging Diagnosis and Radiotherapy, São Paulo State University/UNESP, Medical School, Sao Paulo, Brazil; 3 Department of Gynecology and Obstetrics, São Paulo State University/UNESP, Medical School, Sao Paulo, Brazil; 4 Institute of Health Economics and Clinical Epidemiology, Faculty of Medicine and University Hospital of Cologne, University of Cologne, Cologne, Germany; 5 Department of Health Research Methods, Evidence, and Impact, McMaster University, Hamilton, ON Canada; 6 Biostatistics Unit, St Joseph’s Healthcare-Hamilton, Hamilton, ON Canada; 7 Faculty of Health Sciences, University of Johannesburg, Johannesburg, South Africa

**Keywords:** Chloroquine, Hydroxychloroquine, COVID-19, Adverse effects, Safety, Systematic review

## Abstract

**Background:**

Despite the expectations regarding the effectiveness of chloroquine (CQ) and hydroxychloroquine (HCQ) for coronavirus disease (COVID-19) management, concerns about their adverse events have remained.

**Objectives:**

The objective of this systematic review was to evaluate the safety of CQ and HCQ from malarial and non-malarial randomized clinical trials (RCTs).

**Methods:**

The primary outcomes were the frequencies of serious adverse events (SAEs), retinopathy, and cardiac complications. Search strategies were applied to MEDLINE, EMBASE, LILACS, CENTRAL, Scopus, and Trip databases. We used a random-effects model to pool results across studies and Peto’s one-step odds ratio (OR) for event rates below 1%. Both-armed zero-event studies were excluded from the meta-analyses. We used the Grading of Recommendations Assessment, Development, and Evaluation system to evaluate the certainty of evidence.

**Results:**

One hundred and six RCTs were included. We found no significant difference between CQ/HCQ and control (placebo or non-CQ/HCQ) in the frequency of SAEs (OR: 0.98, 95% confidence interval [CI]: 0.76–1.26, 33 trials, 15,942 participants, moderate certainty of evidence). However, there was a moderate certainty of evidence that CQ/HCQ increases the incidence of cardiac complications (RR: 1.62, 95% CI: 1.10–2.38, 16 trials, 9908 participants). No clear relationship was observed between CQ/HCQ and retinopathy (OR: 1.63, 95% CI: − 0.4–6.57, 5 trials, 344 participants, very low certainty of evidence).

**Conclusions:**

CQ and HCQ probably do not increase SAEs, with low frequency of these adverse events on malarial and non-malarial conditions. However, they may increase cardiac complications especially in patients with COVID-19. No clear effect of their use on the incidence of retinopathy was observed.

**Systematic review registration:**

PROSPERO CRD42020177818

**Supplementary Information:**

The online version contains supplementary material available at 10.1186/s13643-021-01835-x.

## Background

Chloroquine (CQ) and hydroxychloroquine (HCQ) were originally developed for the treatment of malaria; however, several additional drug properties have been discovered, allowing for their use in the treatment of different non-malarial conditions, including rheumatological, dermatological, and immunological diseases [1]. There is also a growing body of evidence to support their therapeutic potential in cancer, chronic kidney disease, and metabolic disorders [1, 2].

The in vitro antiviral activity of CQ has been studied for many decades and the growth of different viruses can be inhibited in vitro by both CQ and HCQ [3]. Therefore, the effectiveness of these drugs has been studied in relation to a variety of acute infectious diseases, including Zika, influenza A H5N1, Ebola, dengue, and chikungunya, as well as chronic viral infections, including hepatitis C and human immunodeficiency virus [3–5].

The most common adverse events (AEs) of these medications are related to gastrointestinal intolerance, such as vomiting, nausea, diarrhea, and abdominal discomfort [6]. Cutaneous manifestations, such as itching, skin rash, photosensitivity, and hyperpigmentation [7] can also occur. Less frequent adverse effects, such as myopathy, neuromyopathy, and cardiotoxicity can be more severe and irreversible [1].

Cardiac conduction disorders (bundle branch block and atrioventricular block), heart failure, ventricular hypertrophy, hypokinesia, valve dysfunction, pulmonary hypertension, and QT prolongation are side effects associated with CQ and HCQ [8].

A long-term AE associated with these medications is retinopathy, which can cause irreversible visual damage. The risk of development is 1% after 5 years of chronic use, which increases to 2 and 20% when used for more than 10 and 20 years, respectively [9].

Faced with the health crisis triggered by the coronavirus disease (COVID-19) pandemic and the absence of a specific drug therapy so far, CQ and HCQ have been evaluated for their possible effectiveness in the treatment of this disease [10]. Nevertheless, despite the expectations regarding their effectiveness, the concern about their adverse side effects has remained. Some researchers consider that a wide use of the drugs will expose some patients to rare but potentially fatal side effects [11], and those who believe in their potential efficacy justify that these medications have a well-established safety profile.

Therefore, as these drugs have been used for many decades for malarial and non-malarial conditions, we conducted a systematic review of randomized clinical trials (RCTs) to evaluate the safety of CQ/HCQ in different conditions and populations.

## Methods

This systematic review was conducted according to Cochrane Collaboration [12] and reported according to Preferred Reporting Items for Systematic Reviews and Meta-Analyses (PRISMA) Statement [13]. Its protocol was registered in the International Prospective Register of Systematic Reviews (CRD42020177818).

### Eligibility criteria

We selected studies that meet the “PICOS” structure described below.

### Participants (P)

An individual, regardless of gender and age, diagnosed with a malarial or non-malarial condition, whose treatment was with either CQ or HCQ.

### Types of interventions (I)

CQ or HCQ.

### Comparison (C)

The comparison group was placebo or no CQ/HCQ. Intervention and comparison groups must have received the same standard treatments for the patient’s basal disease.

### Outcomes (O)

The primary outcomes were the number of patients with serious adverse events (SAEs), the number of patients with retinopathy, and the number of patients with cardiac complications.

We considered any AE or suspected adverse reaction that resulted in any of the following outcomes as SAE: death, a life-threatening AE, hospitalization or prolongation of existing hospitalization, a persistent or significant incapacity, substantial disruption of the ability to conduct normal life functions, or a congenital anomaly/birth defect [14].

We considered retinopathy diagnosed after the use of CQ/HCQ in patients who previously had normal baseline ophthalmologic examination results as CQ/HCQ-induced retinopathy. We considered conduction disorders and other non-specific adverse cardiac events (ventricular hypertrophy, hypokinesia, heart failure, pulmonary arterial hypertension, and valvular dysfunction) as cardiac AE with probable association with CQ or HCQ [8].

The secondary outcomes were the total number of participants with any CQ- or HCQ-related AE, number of withdrawals due to CQ- or HCQ-related AE, number of patients with gastrointestinal AEs (nausea, vomiting, stomach ache, diarrhea, loss of appetite, and weight loss) [1], number of patients with cutaneous manifestations (skin rash, itching, hair loss, erythroderma, exfoliative dermatitis, urticaria, eczematous eruptions, photosensitivity, and erythema annulare centrifugum) [1], number of patients with myopathy, number of patients with visual symptoms, and number of patients with auditory symptoms.

### Study design (S)

We included only RCTs.

### Time of outcome evaluation

The outcomes were evaluated at 4 weeks and after 4 weeks. Trials with outcomes within these time-points were combined with the closest time-point.

### Exclusion criteria

We excluded non-RCTs as well as studies where intervention and comparison groups received different standard treatments for the patient’s basal disease.

### Identification of studies

#### Electronic databases

General search strategies were applied to the main electronic health databases: Embase (Elsevier, 1980–11th April 2020), Medline (PubMed, 1966–11th April 2020), LILACS (Virtual Health Library, 1982–11th April 2020), CENTRAL (Controlled Clinical Trials of Cochrane Collaboration, 1982–11th April 2020), Trip database, SCOPUS, and Web of Science (20th April 2020). A second search on all databases was conducted on 11th April 2021. The search strategies contained index terms and synonyms of chloroquine and hydroxychloroquine. On PubMed, we used the filter for RCT, as supported by Cochrane, and the embedded filter was used for the same purpose on Embase. The search strategy for each database is included in the Supplementary file. References of relevant primary and secondary studies were searched to identify additional eligible studies. As a huge number of studies met our eligibility criteria, the search for unpublished sources (clinical study report, trial registers, and regulatory agency websites) will be performed in a near future.

EndNote X9 citation management software was used to download references and remove duplicate entries. The initial screening of abstracts and titles was performed using the free web application Rayyan QCRI [15].

#### Study selection

Two reviewers (MSB and VSNN) independently selected potentially eligible studies for inclusion in the review based on the titles and abstracts. The studies selected for full-text review were subsequently assessed for adequacy to the proposed ‘PICO’ structure. In case of disagreement, there was a consensus meeting between the reviewers for a final decision.

#### Data extraction and management

The two reviewers (MSB and VSNN) independently used a standard form to extract the following data from the selected studies: year of publication, country, pragmatic or non-pragmatic RCT, basal disorder, population group (children, adults, and pregnant women), sample size, follow-up time, type of intervention and comparison, daily dosage of the intervention, number of patients randomized to intervention and comparison group, number of patients in each group with the primary and secondary outcomes, and mean age of participants.

For a specific outcome, we only extracted data as “zero” if it was clearly listed as such in the study report; otherwise, we interpreted that the authors did not evaluate this outcome.

To ensure consistency between reviewers, we performed a calibration exercise before beginning the review. In the case of duplicate publications or multiple reports from the primary study, data extraction was optimized using the best information available for all items in the same study.

#### Assessment of risk of bias in included studies

For the primary outcomes from each selected trial, the risk of bias was assessed according to the revised Cochrane risk-of-bias tool for RCTs (RoB 2 tool) [16], which considered five domains for each outcome evaluated. The domains were (1) bias arising from the randomization process, (2) bias due to deviations from intended interventions, (3) bias due to missing outcome data, (4) bias in the measurement of the outcome, (5) bias in the selection of the reported result. For cluster randomized trials we used a specific Rob2 tool for cluster RCTs. Each of the items was evaluated in pairs and independently by 14 reviewers as having “low risk of bias,” “some concerns,” or “high risk of bias” on 10th August 2020 and 8th June 2021. We classified SAEs as the outcome available for all or nearly all participants, with less than 5% loss to follow-up. For studies in which such losses were higher than 5%, we considered a low risk of bias in this domain if the rates were balanced between groups, the causes were justified, and not related with any SAE. For outcomes where assessors were aware of the intervention received by study participants, we considered that the assessment of the retinopathy and cardiac complications could have been influenced by the knowledge of the intervention received, but not for SAE. For cardiac complications in open-label studies where there was no information that all participants were subjected to the same method and frequency of investigation, we considered that the measurement or ascertainment of the outcomes might be different between the groups.

#### Unit of analysis

The unit of analysis was the data published in the studies included. We used the data available in published articles, and we preferentially used data from intention-to-treat analysis. For the studies that did not provide an intention to treat analysis, we considered the number of patients randomized in each group, and for patients who missed the follow-up, we input as absent the AE evaluated [17]. For the multi-arm trials, we only selected the groups in which the intervention (HCQ/CQ) and the control (non-intervention or placebo) received the same standard treatments for the patient’s basal disease. For the multi-arm trials with different regimen doses, we combined the groups in which CQ/HCQ were administered.

For the cluster randomized trials, we used a formula suggested by the Cochrane handbook to find the trial’s effective sample size, which is its original sample size divided by the “design effect.” The design effect can be calculated by 1 + (*M* − 1) × ICC, where *M* is the average cluster size and ICC is the intracluster correlation coefficient [12].

#### Data analyses

Similar outcomes were plotted in the meta-analysis using the Stata Statistical Software 17 (Stata Statistical Software: Release 17. College Station, TX: StataCorp LLC). The relative risk (RR) was calculated with a 95% confidence interval (CI) as an effect size of CQ/HCQ, and a random-effect model was used for the meta-analysis. However, as this systematic review involved safety measures, and to avoid underestimating the harm, we used Peto’s one-step odds ratio (OR) method for event rates below 1% [12, 18]. In this situation, both-armed zero-event (BAOE) studies were excluded from the meta-analysis.

#### Sensitivity analysis

For SAE, we performed sensitivity analyses according to the risk of bias (“high risk” versus “some concerns” versus “low risk”), according to the comparison group (placebo versus no CQ/HCQ), and sample size (≥ 100 participants versus < 100 participants). For SAE and cardiac complications, we added per-protocol analyses.

#### Subgroup analysis

For SAE, we performed subgroup analyses according to the type of intervention (CQ or HCQ), patient diagnosis, type of population (children, adults, and pregnant women), daily dosage (< 500 mg versus ≥ 500 mg for CQ, < 400 mg versus ≥ 400 mg for HCQ), time of follow up (≤ 4 weeks versus > 4 weeks). We used the instrument to assess the Credibility of Effect Modification Analyses (ICEMAN tool) to assess the credibility of the subgroups [19].

#### Heterogeneity assessment

Inconsistencies between the results of the studies included were ascertained by visual inspection of forest plots (no overlap of CIs around the effect estimates of the individual studies) and by Higgins or *I*^2^ statistic, in which *I*^2^ > 50% indicated a moderate probability of heterogeneity, and by chi-squared tests (Chi^2^), where *p* < 0.10 indicated heterogeneity.

#### Quality of evidence

The quality of the evidence of the effect size was assessed according to the Grading of Recommendations Assessment, Development, and Evaluation (GRADE) guidelines [20]. GRADE is a structured process for rating the quality of evidence in systematic reviews or in guidelines for health care [21]. Randomized controlled trials begin as high-quality evidence; however, the confidence in the evidence may decrease if the studies have major limitations that may interfere with the estimates of the treatment effect [21]. These limitations include the risk of bias, inconsistency of results, indirectness of evidence, imprecision, and reporting bias [22].

## Results

**Fig. 1:**
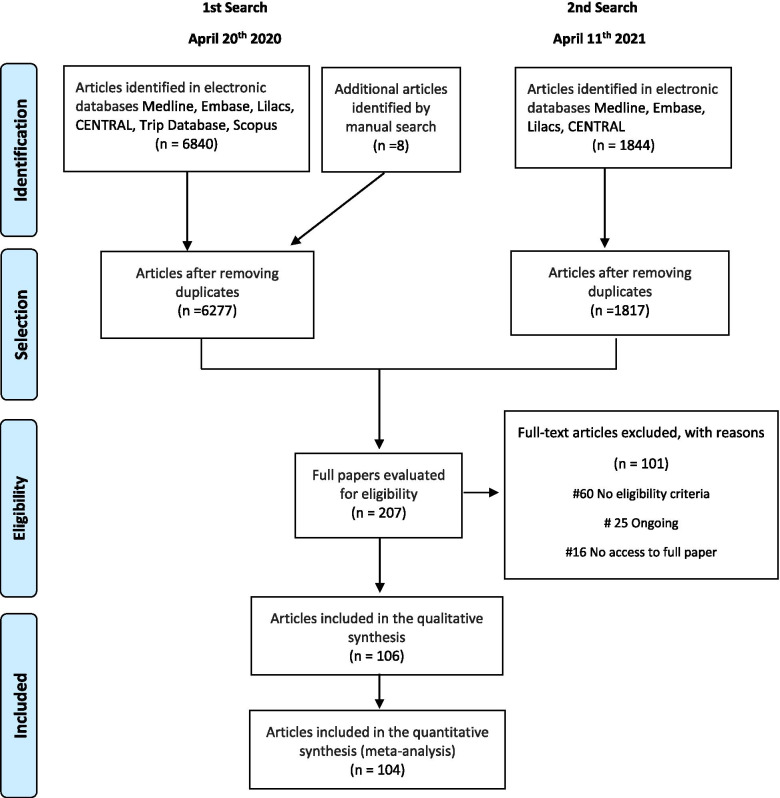
Flow diagram of selected studies

The search strategies yielded different studies, and after removing duplicates, 8094 studies remained. We selected 207 studies that had a high probability of meeting our inclusion criteria for a complete examination (Fig. 1). After completely examining these references, 106 studies met our eligibility criteria and therefore were included in this review.

A total of 101 studies were excluded for the following reasons: no AE was evaluated (*n* = 40), no control group as placebo or non-comparator for CQ or HCQ (*n* = 9), non-RCT (*n* = 5), no report of the outcomes per group studied (*n* = 6) (Supplementary file), studies are still ongoing (*n* = 25) (Supplementary file), and unevaluated eligibility criteria (*n* = 16) (Supplementary file).

### Study characteristics

Out of 106 studies included, 20 were on COVID-19 [23–42], 13 studies were on malaria [43–55], 11 on other infectious conditions [56–67], 31 were on rheumatology [68–98], four on dermatologic diseases [99–102], eight on cancer [103–110], seven were on metabolic disease [111–117], and the remaining were on other conditions [118–128]. Seventy-one studies used HCQ (17,911 participants) as intervention and 35 used CQ (6997 participants). Most studies used placebo as a comparator, seven studies were on children [43, 44, 46, 49, 52, 70, 106], two on pregnant women [55, 90], and the others were on the adult population. Most studies used daily intervention doses ≥ 500 mg for CQ and ≥ 400 mg for HCQ. Rajasinghan et al.’s trial was the only multi-arm trial whose groups received different doses of the same intervention [39]. We combined the groups in which HCQ were administered. We included one cluster randomized trial, Mitjà et al, in the meta-analyses [37].

In most of the included studies, the participants had chronic conditions, and, consequently, the intervention and follow-up were beyond 4 weeks. Meanwhile, the total sample size was higher in studies on acute conditions, with follow-up less than 4 weeks. Table 1 presents descriptive data of all the studies included.

**Table 1:** Data extracted from included studies

**Author**	**Year**	**Country**	**Multicenter**	**Study design**	**Population**	**Condition**	**Intervention**	**Comparator**	**Dosage ≥ 400 mg/day**	**Treatment time > 4weeks**	**Randomized patients**
Type of intervention: Hydroxychloroquine (HCQ)											
**Achuthan**	2014	India	No	RCT crossover	Adult	Other	HCQ	No CQ/HCQ	Yes	No	8
**Abella**	2021	USA	Yes	RCT	Adult	COVID-19	HCQ	Placebo	Yes	Yes	132
**Barnabas**	2021	USA	Yes	RCT	Adult	COVID-19	HCQ	Placebo	Yes	No	829
**Blackburn**	1995	USA	Yes	RCT	Adult	Rheumatology	HCQ	Placebo	Yes	Yes	242
**Bonfante**	2008	Brazil	No	RCT	Adult	Rheumatology	HCQ	Placebo	Yes	Yes	32
**Boonpiyathad**	2017	Thailand	No	RCT	Adult	Dermatology	HCQ	Placebo	Yes	Yes	55
**Boulware**	2020	USA, Canada	Yes	Pragmatic	Adult	COVID-19	HCQ	Placebo	Yes	No	821
**Brazil**	2020	UK	Yes	RCT	Elderly	Oncology	HCQ	Placebo	Yes	Yes	54
**Brewer**	1986	USA, URSS	Yes	RCT	Children	Rheumatology	HCQ	Placebo	Variable	Yes	108
**Bunch**	1984	USA	No	RCT	Adult	Rheumatology	HCQ	Placebo	Variable	Yes	38
**Cavalcanti**	2020	Brazil	Yes	RCT	Adult	COVID-19	HCQ	No CQ/HCQ	Yes	No	448
**Chakravarti**	2021	India	No	RCT	Adult	Metabolic disease	HCQ	placebo	Yes	Yes	326
**Charous**	1998	USA	No	RCT	Adult	Others	HCQ	Placebo	Variable	Yes	17
**Chen Jun**	2020	China	No	RCT	Adult	COVID-19	HCQ	No CQ/HCQ	Yes	No	30
**Chen Zhaowei**	2020	China	No	RCT	Adult	COVID-19	HCQ	Placebo	Yes	No	62
**Cheng-Pin Chen**	2020	China	Yes	RCT	Adult	COVID-19	HCQ	No CQ/HCQ	Yes	No	33
**Clark**	1993	Mexico	No	RCT	Adult	Rheumatology	HCQ	Placebo	Yes	Yes	126
**Das**	2007	India	Yes	RCT	Adult	Rheumatology	HCQ	Placebo	Yes	Yes	122
**Davis**	1991	UK	No	RCT	Adult	Rheumatology	HCQ	Placebo	Yes	Yes	104
**Desta**	2002	Ethiopia	No	RCT	Adult	Others	HCQ	Placebo	No	Yes	61
**Dubée**	2021	France	Yes	RCT	Adult	COVID-19	HCQ	Placebo	Yes	No	250
**Erkan**	2018	USA, Greece, China	Yes	RCT	Adult	Rheumatology	HCQ	No CQ/HCQ	Yes	Yes	20
**Esdaile**	1995	Canada	Yes	RCT	Adult	Rheumatology	HCQ	Placebo	Yes	Yes	119
**Faarvang**	1993	Denmark	Yes	RCT	Adult	Rheumatology	HCQ	Placebo	No	Yes	60
**Fong**	2007	USA	No	RCT	Adult	Others	HCQ	Placebo	Yes	Yes	102
**Gerstein**	2002	Canada	No	RCT	Adult	Metabolic disease	HCQ	Placebo	Yes	Yes	135
**Gilman**	2012	USA	Yes	RCT	Children	Oncology	HCQ	Placebo	Variable	Yes	54
**Gottenberg**	2014	France	Yes	Pragmatic	Adult	Rheumatology	HCQ	Placebo	Yes	Yes	120
**Haar**	1993	Denmark	No	RCT	Adult	Rheumatology	HCQ	Placebo	No	Yes	52
**Horne**	2020	UK/France	Yes	RCT	Adult	Oncology	HCQ	No CQ/HCQ	Yes	Yes	62
**Johnston**	2021	USA	Yes	RCT	Adult	COVID-19	HCQ	Placebo	Yes	No	154
**Jokar**	2013	Iran	No	RCT	Adult	Rheumatology	HCQ	Placebo	Yes	Yes	51
**Karasic**	2019	USA	Yes	RCT	Adult	Oncology	HCQ	No CQ/HCQ	Yes	No	112
**Kavanaugh**	1997	USA	No	RCT	Adult	Rheumatology	HCQ	Placebo	Yes	Yes	11
**Kingsbury**	2018	England	Yes	Pragmatic	Adult	Rheumatology	HCQ	Placebo	Yes	Yes	248
**Kraak**	1965	The Netherlands	No	RCT	Adult	Rheumatology	HCQ	Placebo	Yes	Yes	49
**Kravvariti**	2020	Greece	No	RCT	Adult	Rheumatology	HCQ	No CQ/HCQ	Yes	Yes	50
**Kruize**	1993	Netherlands	Yes	RCT cross over	Adult	Rheumatology	HCQ	Placebo	Yes	Yes	19
**Lee**	2018	Netherlands	Yes	RCT	Adult	Rheumatology	HCQ	Placebo	Yes	Yes	202
**Levy**	2001	Brazil	No	RCT	Pregnant	Rheumatology	HCQ	Placebo	No information	Yes	20
**Liu**	2019	China	No	RCT	Adult	Others	HCQ	Placebo	Yes	Yes	60
**Lyngbakken**	2020	Norway	Yes	Pragmatic	Adult	COVID-19	HCQ	No CQ/HCQ	Yes	No	53
**Majzoobi**	2018	Iran	No	RCT	Adult	Infectious conditions	HCQ	No CQ/HCQ	Variable	Yes	177
**Mitjà**	2020	Spain	Yes	RCT	Adult	COVID-19	HCQ	No CQ/HCQ	Yes	No	353
**Mitjà**	2021	Spain	Yes	Cluster	Adult	COVID-19	HCQ	No CQ/HCQ	Yes	No	674 (effective sample size)
**Murphy**	1987	UK	No	RCT	Adult	Dermatology	HCQ	Placebo	Yes	Yes	31
**O’Dell**	2002	USA	Yes	RCT	Adult	Rheumatology	HCQ	Placebo	Yes	Yes	113
**Omrani**	2020	Qatar	Yes	RCT	Adult	COVID-19	HCQ	Placebo	Yes	No	304
**Pareek**	2015	India	Yes	RCT	Adult	Metabolic disease	HCQ	Placebo	No	Yes	328
**Paton**	2012	UK	No	RCT	Adult	Infectious conditions	HCQ	Placebo	Yes	Yes	83
**Quatraro**	1990	Italy	No	RCT	Adult	Metabolic disease	HCQ	Placebo	Yes	Yes	38
**Rajasingham**	2020	USA, Canada	Yes	RCT	Adult	COVID-19	HCQ	Placebo	Yes	Yes	1483
**Recovery**	2020	UK	Yes	Pragmatic	Adult	COVID-19	HCQ	No CQ/HCQ	Yes	No	4716
**Reeves**	2004	Australia	No	RCT	Adult	Dermatology	HCQ	Placebo	No information	Yes	21
**Reis**	2021	Brazil	Yes	RCT	Adult	COVID-19	HCQ	Placebo	Yes	No	441
**Roberts**	1988	UK	No	RCT cross over	Adult	Others	HCQ	Placebo	Yes	Yes	9
**Sarzi-Puttini**	2003	Italy	Sim	RCT	Adult	Rheumatology	HCQ	No CQ/HCQ	Yes	Yes	71
**Scott**	1989	England	Yes	RCT	Adult	Rheumatology	HCQ	Placebo	Yes	Yes	101
**Self**	2020	USA	Yes	RCT	Adult	COVID-19	HCQ	Placebo	Yes	No	479
**Skipper**	2020	USA, Canada	Yes	RCT	Adult	COVID-19	HCQ	Placebo	Yes	No	491
**Snook**	1981	USA	No	RCT	Adult	Others	HCQ	No CQ/HCQ	Yes	No	50
**Sperber**	1995	USA	No	RCT	Adult	Infectious conditions	HCQ	Placebo	Yes	Yes	40
**Tang**	2020	China	Yes	RCT	Adult	COVID-19	HCQ	No CQ/HCQ	Yes	No	150
**Toledo**	2021	USA	No	RCT	Adult	Metabolic disease	HCQ	Placebo	Yes	Yes	34
**Ulrich**	2020	USA	Yes	RCT	Adult	COVID-19	HCQ	Placebo	Yes	No	128
**Van Gool**	2001	Netherlands	Yes	RCT	Adult	Others	HCQ	Placebo	Yes	Yes	168
**Van Jaarsveld**	2000	Netherlands	Yes	RCT	Adult	Rheumatology	HCQ	No CQ/HCQ	Yes	Yes	187
**Wasko**	2015	USA	No	RCT	Adult	Metabolic disease	HCQ	Placebo	Yes	Yes	33
**Yokogawa**	2017	Japan	Yes	RCT	Adult	Rheumatology	HCQ	Placebo	Yes	Yes	103
**Yoon**	2016	Korea	No	RCT	Adult	Rheumatology	HCQ	Placebo	No	Yes	39
**Zeh**	2020	USA	No	RCT	Adult	Oncology	HCQ	No CQ/HCQ	Yes	Yes	98
**Author**	**year**	**Country**	**Multicenter**	**Study design**	**Population**	**Condition**	**Intervention**	**Comparator**	**Dosage ≥ 500 mg/day**	**Treatment time > 4 weeks**	**Randomized patients**
Type of intervention: Chloroquine (CQ)											
**Arnaout**	2019	Canada	No	RCT	Adult	Oncology	CQ	Placebo	Yes	Variable (2–6 weeks)	70
**Briceno**	2003	Mexico	No	RCT	Adult	Oncology	CQ	No CQ/HCQ	No	Yes	18
**Cox**	2013	Gambia	No	Cluster	Children	Malaria	CQ	Placebo	Variable	Yes	96
**Dunyo**	2006	Gambia	No	RCT	Children	Malaria	CQ	No CQ/HCQ	Variable	No	374
**Endy**	2019	USA	No	RCT	Adult	Malaria	CQ	No CQ/HCQ	Yes	Yes	50
**Engchanil**	2006	Thailand	No	RCT	Children	Infectious conditions	CQ	No CQ/HCQ	Variable	Yes	46
**Fernando**	2006	Sri Lanka	No	RCT	Children	Malaria	CQ	Placebo	No	Yes	587
**Ferraz**	1994	Brazil	Yes	RCT	Adult	Rheumatology	CQ	Placebo	No	Yes	82
**Freedman**	1960	UK	No	RCT	Adult	Rheumatology	CQ	Placebo	No	Yes	107
**Fryauff**	1995	Indonesia	No	RCT	Adult	Malaria	CQ	Placebo	No	Yes	78
**Galatas**	2017	Mozambique	No	RCT	Adult	Malaria	CQ	Placebo	Variable	No	112
**Gasasira**	2003	Uganda	No	RCT	child/adult	Malaria	CQ	Placebo	Variable	No	341
**Gibson**	1987	UK	No	RCT	Adult	Rheumatology	CQ	No CQ/HCQ	No	Yes	52
**Jacobs**	1963	USA	No	RCT	Adult	Dermatology	CQ	Placebo	No	Yes	50
**Jacobson**	2016	USA	No	RCT cross over	Adult	Infectious conditions	CQ	Placebo	No	Yes	70
**Kamgno**	2010	Cameroon	No	RCT	Adult	Infectious conditions	CQ	Placebo	Yes	Yes	40
**Lamballerie**	2008	France	No	RCT	Adult	Infectious conditions	CQ	Placebo	Yes	Yes	54
**McGill**	2019	USA	No	RCT	Adult	Metabolic disease	CQ	Placebo	No	Yes	116
**Meinão**	1996	Brazil	No	RCT	Adult	Rheumatology	CQ	Placebo	No	Yes	24
**Michel**	2010	France	No	RCT	Adult	Malaria	CQ	Placebo	No	Yes	1010
**Miller**	2013	USA	No	RCT	Adult	Malaria	CQ	Placebo	Yes	No	38
**Miranda**	2004	Mexico	Yes	RCT	Adult	Rheumatology	CQ	Placebo	No	Yes	149
**Ndyomugyenyi**	2004	Uganda	No	RCT	Children/aults	Malaria	CQ	No CQ/HCQ	Variable	No	88
**Pappaioanou**	1986	USA	No	RCT	Adult	Infectious conditions	CQ	No CQ/HCQ	No	Yes	51
**Parrow**	1967	Sweden	No	RCT	Adult	Other	CQ	Placebo	No	Yes	18
**Paton**	2011	Singapore	No	RCT	Adult	Infectious conditions	CQ	Placebo	No	Yes	1516
**Peymani**	2016	Iran	No	RCT	Adult	Infectious conditions	CQ	Placebo	No	Yes	12
**Rojas-puentes**	2013	Mexico	No	RCT	Adult	Oncology	CQ	Placebo	No	No	76
**Salako**	1992	Nigeria	No	RCT	Adult	Malaria	CQ	Placebo	No	Yes	229
**Soltani**	2007	Iran	No	RCT	Adult	Others	CQ	Placebo	No	Yes	24
**Sotelo**	2006	Mexico	No	RCT	Adult	Oncology	CQ	Placebo	No	Yes	30
**Terrabuio**	2018	Brazil	No	RCT	Adult	Others	CQ	Placebo	No	Yes	62
**Tricou**	2010	Vietnam	No	RCT	Adult	Infectious conditions	CQ	Placebo	Yes	No	307
**Vicente**	2019	USA	No	RCT	Adult	Malaria	CQ	Placebo	Yes	No	20
**Villegas**	2007	Thailand	No	RCT	Pregnant	Malaria	CQ	Placebo	No	Yes	1000

*Legend*: *HCQ* hydroxychloroquine, *CQ* chloroquine, *RCT* randomized controlled trial

### Risk of bias

**Fig. 2:**
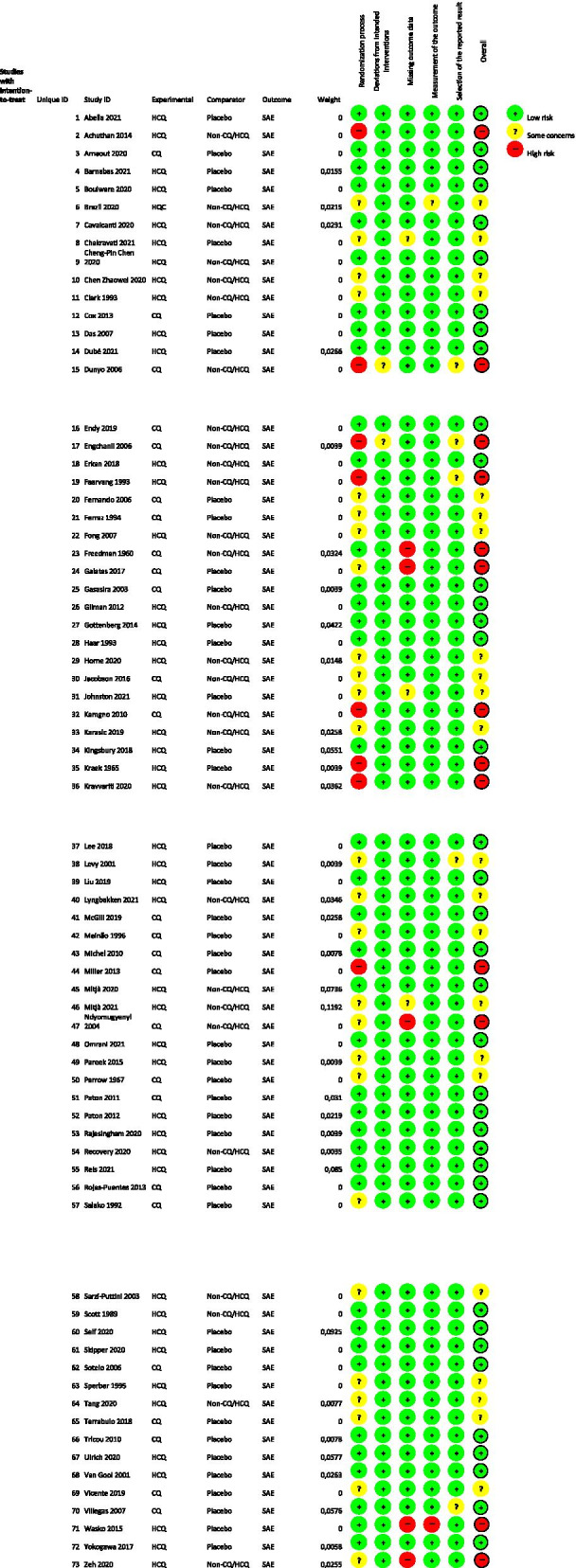
Risk of bias according to RoB 2 for serious adverse events (SAE)

Figure 2 shows the risk of bias corresponding to the included studies for the SAE outcome, and Fig. XIX and Fig. XX in the Supplementary file, respectively, present the risk of bias for retinopathy and cardiac complications. Regarding SAEs, most studies were assessed as low risk of bias, while some were classified as some concerns or high risk due mainly to randomization process or missing outcome data. For retinopathy, most studies did not mention the method used to evaluate this outcome, and because of that, they were classified mostly as some concerns or high risk of bias. For cardiac complications, the open-label RCTs were graduated as some concerns of risk of bias due to no information if participants of both groups were submitted to the same method and frequency of the outcome evaluation.

### Meta-analysis

**Fig. 3:**
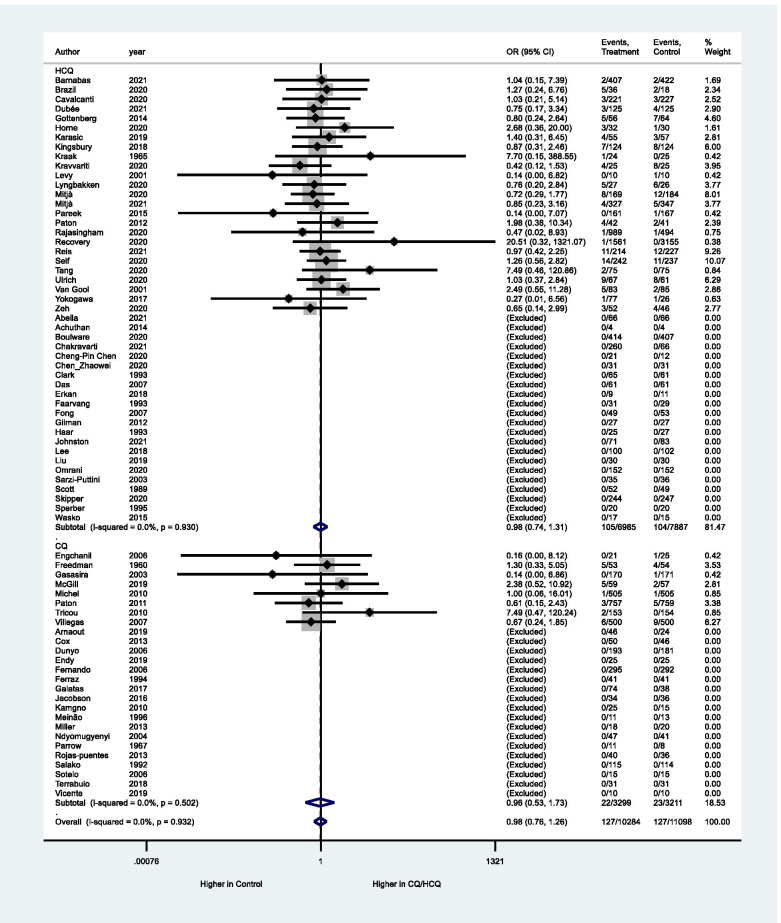
Meta-analysis for frequency of serious adverse events. Subgroup analysis according to the type of intervention. CQ, chloroquine; HCQ, hydroxychloroquine. Peto’s method was adopted and studies with both-armed zero-event were excluded from the analysis. Significance test(s) of OR = 1, overall, *z* = 0.16, *p* = 0.872

Regarding primary outcomes, there is no evidence to support the difference between CQ/HCQ and the control group (placebo or non-CQ/HCQ) with regard to the frequency of SAE (OR: 0.98, 95% CI: 0.76–1.26, 33 studies, 15,942 participants, moderate certainty of evidence, Table 2, Fig. 3). Forty BAOE studies with 5440 participants were excluded from this analysis (Fig. 3). Applying the GRADE approach, due to non-inclusion of unpublished data, the quality of evidence was rated down in one level.

**Table 2:** Summary of findings according to GRADE approach. CQ/HCQ compared to Placebo or no CQ/HCQ for malarial and non-malarial conditions

Outcomes	Anticipated absolute effects^*^ (95% CI)		Relative effect (95% CI)	№ of participants (studies)	Certainty of the evidence (GRADE)	Comments
	Risk with placebo/non-comparator	Risk with HCQ/CQ				
SAE	14 per 1.000	**15 per 1.000** (12 to 20)	**OR 0.98** (0.76 to 1.26)	15942 (33 RCTs)	⨁⨁⨁◯ MODERATE ^a^	CQ/HCQ likely does not increase SAE.
Retinopathy	18 per 1.000	**28 per 1.000** (7 to 105)	**OR 1.63** (0.40 to 6.57)	344 (5 RCTs)	⨁⨁◯◯ LOW ^b^	The evidence is very uncertain about the effect of CQ/HCQ on retinopathy.
Cardiac Complications	20 per 1.000	**37 per 1.000** (25 to 55)	**RR 1.62** (1.10 to 2.38)	9908 (16 RCTs)	⨁⨁⨁◯ MODERATE ^c^	CQ/HCQ may result in an increase in cardiac complications.

*The risk in the intervention group (and its 95% confidence interval) is based on the assumed risk in the comparison group and the relative effect of the intervention (and its 95% CI). *CI* confidence interval, *OR* odds ratio, *RR* risk ratio, *SAE* serious adverse events, *CQ* chloroquine; *HCQ* hydroxychloroquine, *GRADE* Grading of Recommendations Assessment, Development, and Evaluation

GRADE levels of evidence

High certainty: We are very confident that the true effect lies close to that of the estimate of the effect

Moderate certainty: We are moderately confident in the effect estimate: The true effect is likely to be close to the estimate of the effect, but there is a possibility that it is substantially different

Low certainty: Our confidence in the effect estimate is limited: The true effect may be substantially different from the estimate of the effect

Very low certainty: We have very little confidence in the effect estimate: The true effect is likely to be substantially different from the estimate of effect

Explanations

^a^The quality of evidence was rated down due to non-inclusion of unpublished data

^b^Considering a prevalence of 7.8% of retinopathy in non-diabetic population (Klein,1992), and a 1% of retinopathy risk in the first 5 years of HCQ treatment (Petri 2020), the minimum sample size required for detection of this outcome is 4533 (level of significance =5%; power=1- β=80%). Then, the optimal information size criterion was not met, and the confidence interval was wide. Because of this the quality of evidence was rating downed for imprecision

^c^The open-label RCTs were graduated as some concerns of risk of bias due to no information if participants of both groups were submitted to the same method and frequency of the outcome evaluation

**Fig. 4:**
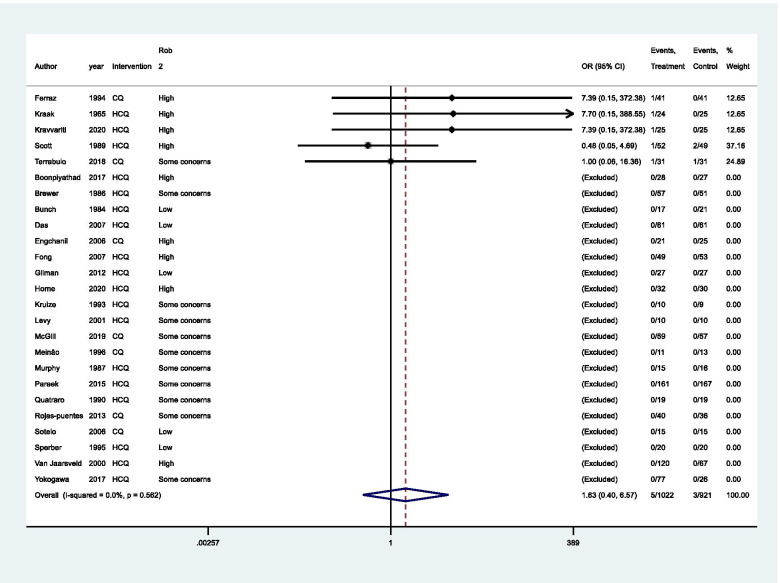
Meta-analysis for frequency of retinopathy. CQ, chloroquine; HCQ, hydroxychloroquine. Peto’s method was adopted and studies with both-armed zero-event were excluded from the analysis. Significance test(s) of OR = 1, *z* = 0.69, *p* = 0.492

Regarding the association between CQ/HCQ and the frequency of retinopathy, the evaluation of the risk of bias and imprecision (wide confidence interval, no achievement of optimal information size) did not indicate any clear effect (OR: 1.63, 95% CI: − 0.4–6.57, 5 studies, 344 participants, very low certainty of evidence, Fig. 4, Table 2). Twenty BAOE studies with 1559 participants were excluded from this analysis (Fig. 4).

**Fig. 5:**
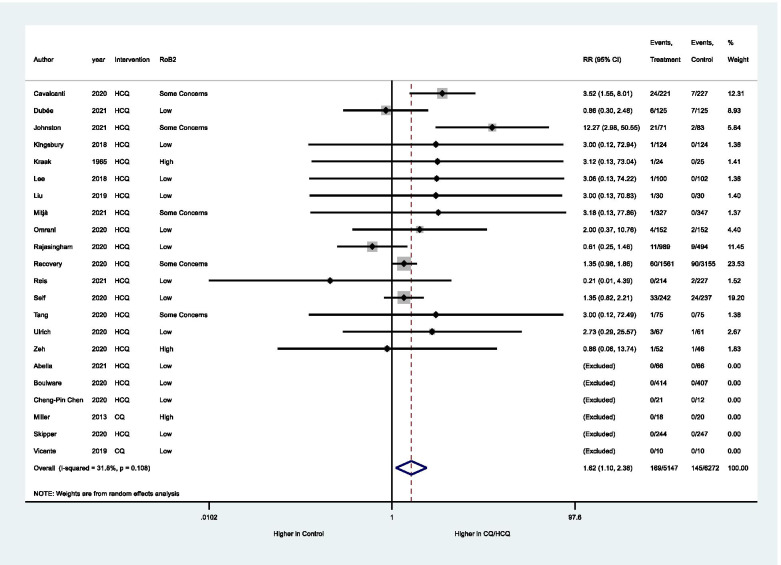
Meta-analysis for frequency of cardiac complications. Studies with both-armed zero-event were excluded from the analysis. Significance test(s) of RR = 1, *z* = 2.60, *p* = 0.009

The meta-analysis showed that HCQ increases the incidence of cardiac complications (RR: 1.62, 95% CI: 1.1–2.38, 16 trials, 9908 participants, moderate certainty of evidence, Fig. 5, Table 2), six BAOE studies were excluded from this analysis. Out of the 16 RCTs included in this meta-analysis, 11 were in COVID-19 patients (10,390 participants). Applying Rob2, the open-label RCTs were evaluated considering some concerns about the risk of bias in the measurement of this outcome. It occurred because there is no information if participants of both groups were submitted to the same method and frequency of the evaluation of cardiac complications. Consequently, the quality of evidence was rated down. The complications reported were cardiac arrhythmia and prolongation of QTc interval. Two studies reported these complications as SAE (a prolonged QT interval with ventricular arrhythmias, and a case of torsades de pointes) [26, 85]. The sensitivity analysis per protocol did not change the effect size of HCQ in these outcomes (Supplementary file).

For the secondary outcomes, the administration of CQ/HCQ increases the incidence of total AE (RR 1.45, 95% CI: 1.26–1.69, 51 studies, 13,034 participants, Supplementary file), nausea/vomiting (RR 1.93 95% CI: 1.51–2.49, 26 studies, 7981 participants, Supplementary file), diarrhea (RR 2.04% CI: 1.41–2.93, 23 studies, 8378 participants, Supplementary file), withdrawal due to AE (RR 1.38, 95% CI: 1.12–1.71, 41 studies, 7472 participants, Supplementary file), auditory symptoms (RR 1.82, 95% CI: 1.09 to 3.03, 9 studies, 5199 participants) and dermatological affections (RR 1.62, 95% CI: 1.18–2.23, 20 studies, 7026 participants, Supplementary file). There was no clear evidence to support a difference between the CQ/HCQ and control group with regard to visual symptoms and headache (RR 1.59, 95% CI: 1.00 to 2.54, 26 studies, 9210 participants; RR 1.47, 95% CI: 1.02–2.13, 29 studies, 9953 participants, respectively, Supplementary file). Only two studies reported myopathy as AE, and no difference was found between the groups.

For SAE, although more than ten studies were included in the meta-analysis, we could not evaluate publication bias by funnel plot or Egger test because all the studies showed no statistically significant difference between groups (CQ/HCQ versus placebo or non-CQ/HCQ). Nevertheless, the quality of evidence in this domain was rated down due to the non-inclusion of unpublished data.

The subgroup analysis according to the type of intervention, patient diagnosis, type of population, daily dosage, and time of follow-up did not indicate that CQ/HCQ increased the frequency of SAE (Supplementary file). However, for the subgroup analysis of studies (not for subsets of participants), with 3–4 studies in the smallest subgroup, all the effect modification analyses were classified as having low credibility.

In the sensitivity analyses (according to overall risk of bias, placebo or non-CQ/HCQ, sample size), a “no true” CQ/HCQ effect on SAE was observed (Supplementary file).

## Discussion

Considering the promising action of CQ and HCQ in the treatment of COVID-19 at the beginning of the pandemic, many guidelines started to include them in the management of this condition. Consequently, a significant number of patients used these medications, and a great concern emerged regarding their safety. Thus, we conducted a systematic review of RCTs to evaluate the safety of CQ/HCQ in different conditions and populations.

We chose, as our primary outcomes, the frequency of rare but potentially fatal AEs: SAEs, retinopathy, and cardiac complications. The study included 106 RCTs, with a total of 24,879 participants. With moderate certainty, we did not find evidence that either CQ or HCQ, compared with placebo or non-CQ/HCO, increased the frequency of SAEs. However, the HCQ increased the incidence of cardiac complications in the trials whose condition was COVID-19. Due to imprecision and risk of bias, we did not observe any clear effect of CQ/HCQ on retinopathy.

Although the literature has reported several CQ/HCQ-associated AEs, both drugs are generally considered safe [129–132]. One of the explanations that HCQ increased the incidence of cardiac complication is that COVID-19 patients have been considered at an increased risk of cardiac arrhythmias [133]. Moreover, some tachyarrhythmias have been observed in this population, being atrial fibrillation, atrial flutter, ventricular tachycardia, and ventricular fibrillation the most frequent [133]. A systematic review under conditions other than COVID-19 identified 86 case/series studies, providing information on 127 participants with cardiac complications likely to be caused by CQ/HCQ. Majority of the patients were treated with CQ and most had been treated for a long time (median, 7 years; minimum, 3 days; maximum, 35 years), with high cumulative doses (median, 1235 g and 803 g for hydroxychloroquine and chloroquine, respectively). Conduction disorders were the main cardiac complication reported, affecting 85% of the patients. Moreover, the authors highlighted that case/series studies do not allow for an association of causality, and the risk of cardiac complications attributed to CQ/HCQ could not be quantified [8].

While this review was being performed, two unregistered systematic reviews were published on the same subject. Ren et al. identified RCTs that compared the safety profiles of CQ or HCQ with placebo or other active treatment. Their study included 40 studies, with 2137 participants and 1096 participants in the CQ and HCQ trials, respectively. They used RR as effect size, and they concluded that the overall mild or total AEs were statistically higher with CQ or HCQ than with placebo. From the meta-analysis of SAEs, the RR values for CQ and HCQ were 1.1 (95% CI: 0.41 to 2.9, six studies) and 1.12 (95% CI: 0.58 to 2.15, 14 trials), respectively [134]. Eljaaly et al. searched PubMed and EMBASE databases for RCTs of adults comparing AE of HCQ with placebo for any indication. Nine RCTs with a total of 916 patients were included. Cardiac toxicity was not reported, and the meta-analysis found a significantly higher risk of skin pigmentation in HCQ users than in those that received placebo. However, the study did not evaluate the frequency of SAE and retinopathy [135].

Our review included more studies and consequently more participants than the two published reviews. Both reviews included only studies with placebo and the latter included only the studies on HCQ. We did not exclude non-placebo-controlled studies in the evaluation of SAE because we did not believe that the lack of blinding in the outcome assessment, as well as in the intervention received, could cause performance or detection bias. Additionally, we performed a sensitivity analysis separating placebo trials from the trials with non-CQ/HCQ, and for each subgroup analysis, there was no difference in the frequency of SAE, and the CIs were the same (Supplementary file).

Our systematic review had some limitations. The most significant was that there was no search for unpublished sources of data on AE. This includes clinical study reports, trial registers, and regulatory agency websites [12]. There is strong evidence that much of the information on AE are unpublished and that the number and range of AE are higher in unpublished than in published versions of the same study [136]. Golder and colleagues found that the median percentage of published documents with AE information was 46% compared with 95% in the corresponding unpublished documents [136]. Because of this, we rated down the quality of evidence by one level for publication bias. Additionally, the search for SAE, retinopathy, and cardiac complications related to CQ/HCQ from unpublished data will be the next step of this project. The second limitation was the number of BAOE studies that were excluded from the meta-analysis of primary outcomes. As these studies do not provide any indication of direction or magnitude of the relative treatment effect, they are naturally excluded in meta-analysis of OR and RR [12]. However, there is no consensus on whether studies with no observed events in the treatment and control arms should be included or not in a meta-analysis of RCTs. Cheng and collaborators simulated 2500 data sets for rare event outcomes with different scenarios by varying the baseline event rate, treatment effect and number of patients in each trial, and between-study variance [18]. In accordance with another study [137], they concluded that the Peto one-step odds ratio method is the least biased and most powerful method for the meta-analyses of rare events [12]. Additionally, including BAOE studies for AE can underestimate possible harmful side effects, which could expose patients to unnecessary danger. Thus, they recommended that for the analysis of rare AEs, Peto’s method should be adopted in conjunction with the exclusion of BAOE studies from analysis. The third limitation was that most of the trials included were not pragmatic studies, and individuals with risk factors for AEs related to CQ/HCQ were excluded. This means that the safety profile of CQ/HCQ presented here was not designed to the real-world, and it could be underestimating harm.

## Conclusion

In conclusion, from the findings of this systematic review, CQ and HCQ probably do not increase the frequency of SAE from RCTs on malarial and non-malarial conditions. However, they may increase cardiac complications in patients with COVID-19. Due to imprecision and bias in the measurement of the outcomes, no clear effect on the incidence of retinopathy was observed.

## Supplementary Information

**Additional file 1.**

## Data Availability

All data generated or analyzed for this systematic review are included in this published article (and its Supplementary file).

## Abbreviations

AE: Adverse event
BAOE: Both-armed zero-event
CI: Confidence interval
CQ: Chloroquine
HCQ: Hydroxychloroquine
OR: Odds ratio
RCT: Randomized controlled trial
RR: Relative risk
SAE: Serious adverse events
