# Supplementary material for: Systematic review and meta-analysis of the safety of chloroquine and hydroxychloroquine from randomized controlled trials on malarial and non-malarial conditions
Source: Syst Rev. 2021 Nov 4;10:294. doi: 10.1186/s13643-021-01835-x (PMC8567984; doi:10.1186/s13643-021-01835-x)
Supplement: Supplementary file 1 — Additional file 1. [file 13643_2021_1835_MOESM1_ESM.docx]

**Supplementary File**

**Systematic review and meta-analysis of the safety of chloroquine and hydroxychloroquine from randomized controlled trials on malarial and non-malarial conditions**

Mayra Souza Botelho^1^, Fernanda Bolfi^1^, Renata Giacomini Occhiuto Ferreira Leite^1^, Mauro Salles Ferreira Leite^1^, Luisa Rocco Banzato^1^, Luiza Teixeira Soares^1^, Thaina Oliveira Felicio Olivatti ^1^, Amanda Sampaio Mangolim^1^, Flávia Ramos Kazan Oliveira^1^, Luciana Patrícia Fernandes Abbade^2^, Joelcio Francisco Abbade^3^, Ricardo Augusto Monteiro de Barros Almeida^2^, Julia Simões Correa Galendi^4^, Lehana Thabane^5,6^, Vania dos Santos Nunes-Nogueira^1^

1. Department of Internal Medicine , São Paulo State University/UNESP, Medical School, Sao Paulo, Brazil
2. Department of Infectious Diseases, Dermatology, Imaging Diagnosis and Radiotherapy, São Paulo State University/UNESP, Medical School, Sao Paulo, Brazil
3. Department of Gynecology and Obstetrics, São Paulo State University/UNESP, Medical School, Sao Paulo, Brazil
4. Institute of Health Economics and Clinical Epidemiology, University Hospital of Cologne, Cologne, Germany
5. Department of Health Research Methods, Evidence, and Impact, McMaster University, Hamilton, ON, Canada.
6. Departments of Pediatrics and Anesthesia, McMaster University, Hamilton, ON, Canada.

Corresponding Author:

Vania dos Santos Nunes Nogueira

Departamento de Clínica Médica – FMB – UNESP

Avenida Professor Mário Rubens Guimarães Montenegro s/n, Bairro UNESP, Campus Botucatu, Botucatu-SP 18618-687, Brazil

Phone: (55 14) 3880 11 71. Fax: (55 14) 3880 16 67

E-mail: [vania.nunes-nogueira@unesp.br](mailto:vania.nunes-nogueira@unesp.br)

Orcid id: <http://orcid.org/0000-0001-9316-4167>

1. **Meta-analyses of secondary outcomes** ----------------------------------------------- **3**

**Figure I.** Meta-analysis of total adverse events **----------------------------------------------------**3

**Figure II.** Meta-analysis of withdrawal due to any adverse event**-------------------------------**4

**Figure III.** Meta-analysis of adverse event nausea and/or vomiting**-----------------------------**5

**Figure IV.** Meta-analysis of adverse event diarrhea **----------------------------------------------**6

**Figure V.** Meta-analysis of adverse events of dermatology affections**--------------------------**7

**Figure VI.** Meta-analysis of adverse event visual complaints**------------------------------------**8

**Figure VII.** Meta-analysis of adverse event headache**---------------------------------------------**9

**Figure VIII**. Meta-analysis of adverse event auditory symptoms**------------------------------**10

1. **Analyses by subgroups – Serious Adverse Events** --------------------------------**11**

**Figure IX.** Subgroup analysis according to the disease **------------------------------------------**11

**Figure X.** Subgroup analysis according to the time of treatment **-------------------------------**12

**Figure XI.** Subgroup analysis according to the dose of the intervention**-----------------------**13

**Figure XII.** Subgroup analysis according to the studied population **---------------------------**14

1. **Sensitivity Analyses - Serious Adverse Events (SAE)** ---------------------------**15**

**Figure XIII.** Sensitivity analysis according to risk of bias (RoB 2) **---------------------------**15

**Figure XIV.** Sensitivity analysis according to blind of participants and personnel **----------**16

**Figure XV.** Sensitivity analysis according to sample size **---------------------------------------**17

**Figure XVI.** Sensitivity analysis per protocol **-----------------------------------------------------**18

1. **Sensitivity Analyses - Cardiac Arrythmia** -----------------------------------------**19**

**Figure XVII.** Meta-analysis of cardiac arrythmia**.** Sensitivity analysis per protocol **--------**19

1. **Risk of bias (RoB 2) Retinopathy (Figure XVIII)** ---------------------------------**20**
2. **Risk of bias (RoB 2) Cardiac complications (Figure XIX)** -----------------------**21**
3. **References of excluded studies** -------------------------------------------------------**22**
4. **References of ongoing studies** --------------------------------------------------------**25**
5. **References of studies we could not have full access** ------------------------------**27**
6. **Search Strategies** -----------------------------------------------------------------------**28**
7. **Meta-analyses of secondary outcomes**

**Fig. I** Meta-analysis of total adverse events. Subgroup analysis according to type of intervention. CQ: Chloroquine, HCQ: Hydroxychloroquine.

**Fig. II** Meta-analysis of withdrawal due to any adverse event**.** Subgroup analysis according to type of intervention. CQ: Chloroquine, HCQ: Hydroxychloroquine. Significance test(s) of RR=1 Overall, p = 0.003.

**Fig. III** Meta-analysis of adverse event nausea and/or vomiting. Subgroup analysis according to type of intervention. CQ: Chloroquine, HCQ: Hydroxychloroquine.

**Fig. IV** Meta-analysis of adverse event diarrhea**.** Subgroup analysis according to type of intervention. CQ: Chloroquine, HCQ: Hydroxychloroquine.

**Fig. V** Meta-analysis of adverse events of dermatology affections. Subgroup analysis according to type of intervention. CQ: Chloroquine, HCQ: Hydroxychloroquine.

**Fig. VI** Meta-analysis of adverse event visual complaints**.** Subgroup analysis according to type of intervention. CQ: Chloroquine, HCQ: Hydroxychloroquine.

**Fig. VII** Meta-analysis of adverse event headache**.** Subgroup analysis according to type of intervention. CQ: Chloroquine, HCQ: Hydroxychloroquine.

**Fig. VIII** Meta-analysis of adverse event auditory symptoms**.** Subgroup analysis according to type of intervention. CQ: Chloroquine, HCQ: Hydroxychloroquine

1. **Analyses by subgroups – Serious Adverse Events**

**Fig. IX** Meta-analysis of serious adverse events. Subgroup analysis according to the disease.

**Fig. X** Meta-analysis of serious adverse events. Subgroup analysis according to the time of treatment. No: prophylaxis and acute conditions which time of treatment was ≤ 4 weeks. Yes: chronic conditions (> 4 weeks).

**Fig. XI** Meta-analysis of serious adverse events. Subgroup analysis according to the dose of the intervention. Yes: daily dose ≥ 500 mg for chloroquine and ≥ 400 mg for hydroxychloroquine. No: daily dose < 500 mg for chloroquine and < 400 mg for hydroxychloroquine.

**Fig. XII** Meta-analysis of serious adverse events. Subgroup analysis according to the studied population: adult, children or pregnant

1. **Sensitivity Analyses - Serious Adverse Events (SAE)**

**Fig. XIII** Meta-analysis of serious adverse events**.** Sensitivity analysis according to risk of bias (RoB 2).

**Fig. XIV** Meta-analysis of serious adverse events**.** Sensitivity analysis according to blind of participants and personnel.

**Fig. XV** Meta-analysis of serious adverse events. Sensitivity analysis according to sample size: Yes: ≥ 100 participants. No: < 100 participants.

**Fig. XVI** Meta-analysis of serious adverse events**.** Sensitivity analysis per protocol.

1. **Sensitivity Analyses - Cardiac Complications**

**Fig. XVII** Meta-analysis of cardiac complications**.** Sensitivity analysis per protocol.

1. **Risk of bias (RoB 2)**

**Fig. XVIII Risk of bias of Retinopathy
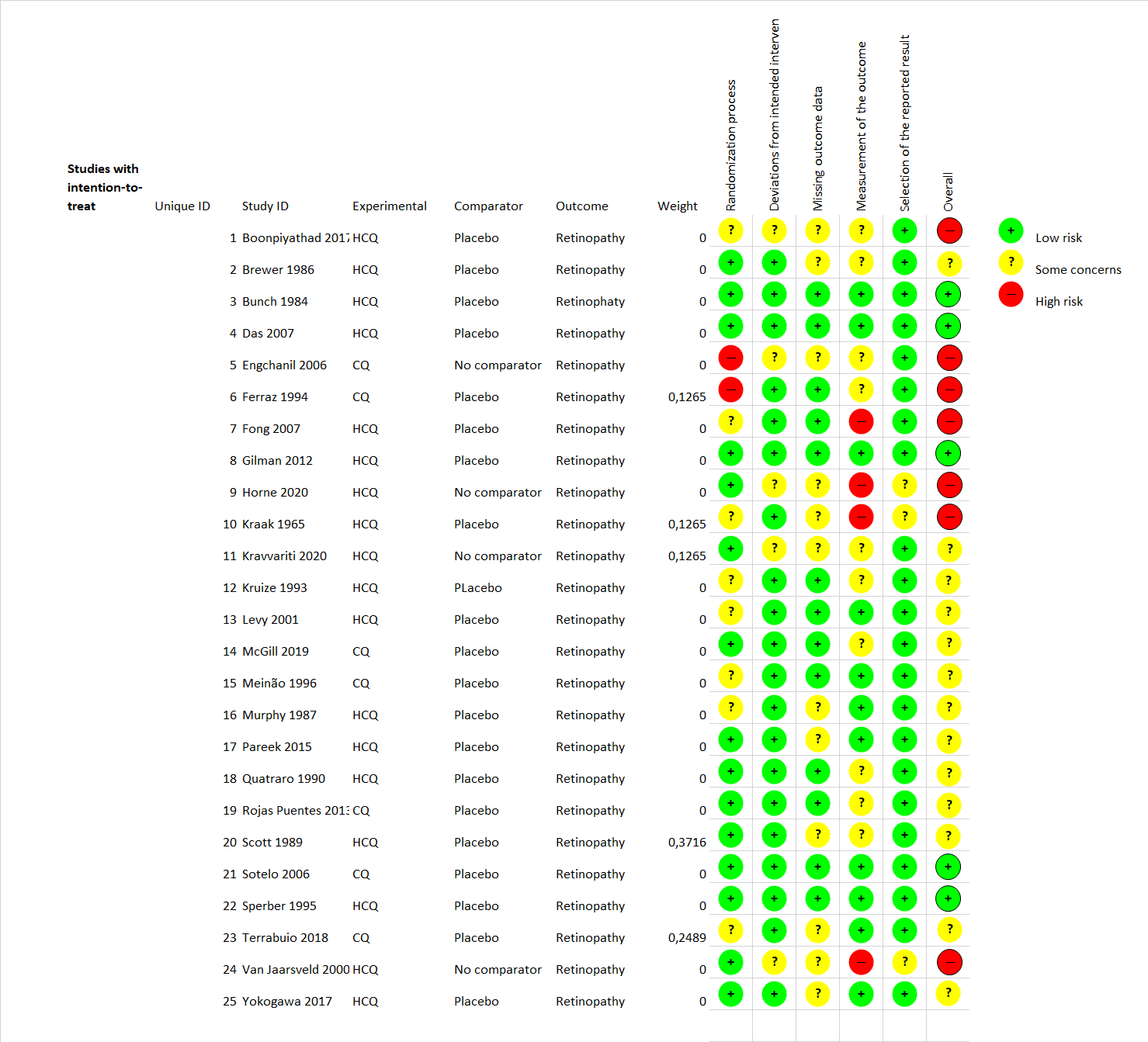
**

1. **Risk of bias (RoB 2)**

**Fig. XIX Risk of bias of Cardiac complication**
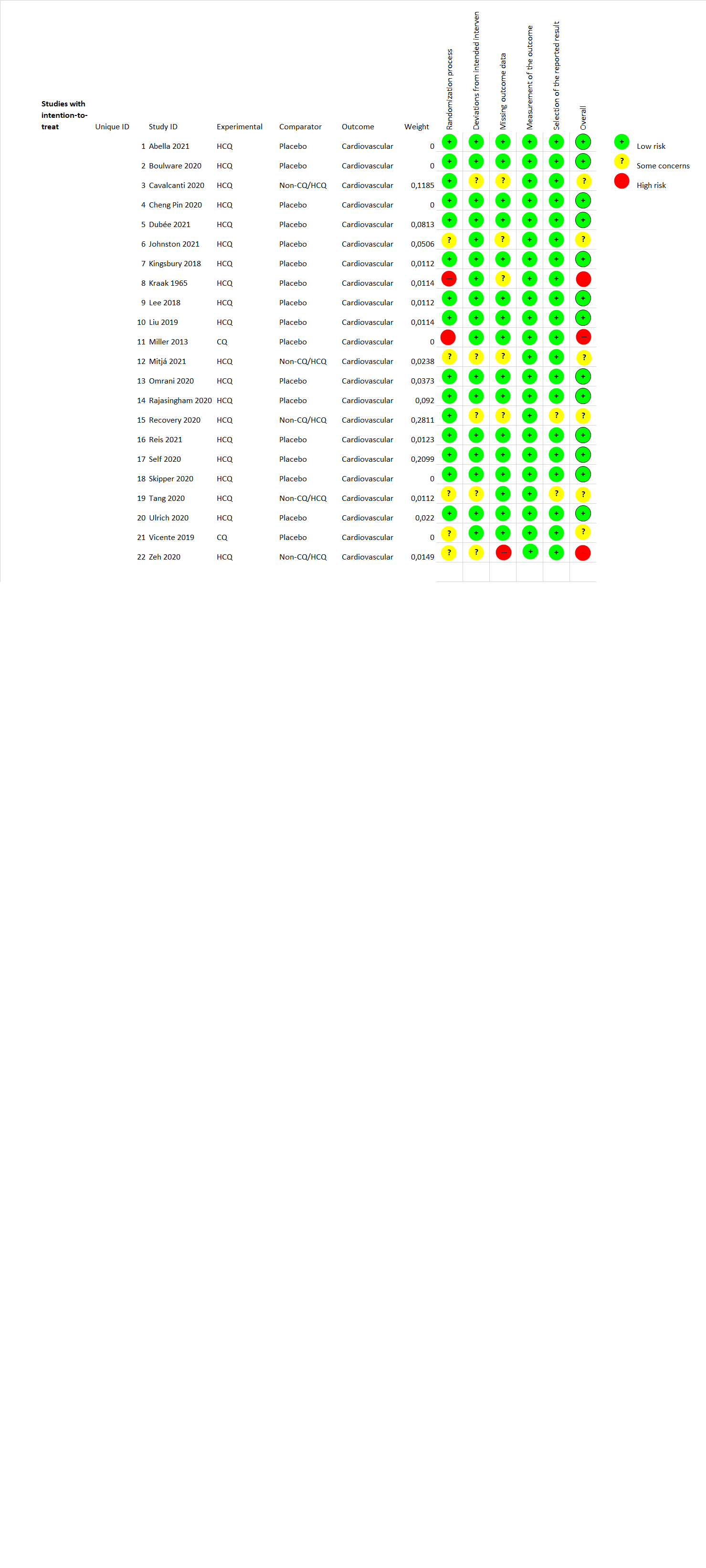


1. **References of excluded studies**

1. Abd-Elsalam S, Esmail ES, Khalaf M, Abdo EF, Medhat MA, Abd El Ghafar MS, et al. Hydroxychloroquine in the Treatment of COVID-19: A Multicenter Randomized Controlled Study. Am J Trop Med Hyg. 2020;103(4):1635-9.

2. Baltzan M, Mehta S, Kirkham TH, Cosio MG. Randomized trial of prolonged chloroquine therapy in advanced pulmonary sarcoidosis. American journal of respiratory and critical care medicine. 1999;160(1):192-7.

3. Björkman A, Brohult J, Pehrson P, Willcox M, Rombo L, Hedman P, et al. Monthly antimalarial chemotherapy to children in a holoendemic area of Liberia. Annals of Tropical Medicine & Parasitology. 1986;80(2):155-67.

4. Blair S, López ML, Piñeros JG, Álvarez T, Tobón A, Carmona J. Eficacia terapéutica de tres esquemas de tratamiento de malaria no complicada por Plasmodium falciparum, Antioquia, Colombia, 2002. Biomédica. 2003;23(3):318-27.

5. Bodewes IL, Gottenberg J-E, van Helden-Meeuwsen CG, Mariette X, Versnel MA. Hydroxychloroquine treatment downregulates systemic interferon activation in primary Sjögren’s syndrome in the JOQUER randomized trial. Rheumatology. 2020;59(1):107-11.

6. Bojang KA, Schneider G, Forck S, Obaro SK, Jaffar S, Pinder M, et al. A trial of Fansidar plus chloroquine or Fansidar alone for the treatment of uncomplicated malaria in Gambian children. Trans R Soc Trop Med Hyg. 1998;92(1):73-6.

7. Borba MGS, Val FFA, Sampaio VS, Alexandre MAA, Melo GC, Brito M, et al. Effect of High vs Low Doses of Chloroquine Diphosphate as Adjunctive Therapy for Patients Hospitalized With Severe Acute Respiratory Syndrome Coronavirus 2 (SARS-CoV-2) Infection: a Randomized Clinical Trial. JAMA network open. 2020;3(4):e208857.

8. Borges MC, Castro LA, Fonseca BA. Chloroquine use improves dengue-related symptoms. Mem Inst Oswaldo Cruz. 2013;108(5):596-9.

9. Calgüneri M, Pay S, Caliskaner Z, Apras S, Kiraz S, Ertenli I, et al. Combination therapy versus monotherapy for the treatment of patients with rheumatoid arthritis. Clin Exp Rheumatol. 1999;17(6):699-704.

10. Carmichael SJ, Beal J, Day RO, Tett SE. Combination therapy with methotrexate and hydroxychloroquine for rheumatoid arthritis increases exposure to methotrexate. J Rheumatol. 2002;29(10):2077-83.

11. Carter A, Eban R. Prevention of postoperative deep venous thrombosis in legs by orally administered hydroxychloroquine sulphate. Br Med J. 1974;3(5923):94-5.

12. Charous BL. Open study of hydroxychloroquine in the treatment of severe symptomatic or corticosteroid-dependent asthma. Ann Allergy. 1990;65(1):53-8.

13. Coosemans MH, Barutwanayo M, Onori E, Otoul C, Gryseels B, Wéry M. Double-blind study to assess the efficacy of chlorproguanil given alone or in combination with chloroquine for malaria chemoprophylaxis in an area with Plasmodium falciparum resistance to chloroquine, pyrimethamine and cycloguanil. Trans R Soc Trop Med Hyg. 1987;81(1):151-6.

14. Costedoat-Chalumeau N, Galicier L, Aumaître O, Francès C, Guern VL, Lioté F, et al. Hydroxychloroquine in systemic lupus erythematosus: Results of a French multicentre controlled trial (PLUS Study). Annals of the Rheumatic Diseases. 2013;72(11):1786-92.

15. Cot M, Le Hesran J-Y, Miailhes P, Roisin A, Fievet N, Barro D, et al. Effect of chloroquine prophylaxis during pregnancy on maternal haematocrit. Annals of Tropical Medicine & Parasitology. 1998;92(1):37-43.

16. De Feo P, Volpi E, Lucidi P, Cruciani G, Santeusanio F, Bolli GB, et al. Chloroquine reduces whole body proteolysis in humans. American Journal of Physiology-Endocrinology and Metabolism. 1994;267(1):E183-E6.

17. Dhibar DP, Arora N, Kakkar A, Singla N, Mohindra R, Suri V, et al. Post-exposure prophylaxis with hydroxychloroquine for the prevention of COVID-19, a myth or a reality? The PEP-CQ Study. Int J Antimicrob Agents. 2020;56(6):106224.

18. Dunne MW, Singh N, Shukla M, Valecha N, Bhattacharyya PC, Dev V, et al. A multicenter study of azithromycin, alone and in combination with chloroquine, for the treatment of acute uncomplicated Plasmodium falciparum malaria in India. J Infect Dis. 2005;191(10):1582-8.

19. Eldredge HB, DeNittis A, DuHadaway JB, Chernick M, Metz R, Prendergast GC. Concurrent whole brain radiotherapy and short-course chloroquine in patients with brain metastases: a pilot trial. Journal of radiation oncology. 2013;2(3):315-21.

20. Fryauff DJ, Richards AL, Baird JK, Richie TL, Mouzin E, Tjitra E, et al. Lymphocyte proliferative response and subset profiles during extended periods of chloroquine or primaquine prophylaxis. Antimicrobial agents and chemotherapy. 1996;40(12):2737-42.

21. Fryauff DJ, Church LP, Richards AL, Widjaja H, Mouzin E, Ratiwayanto S, et al. Lymphocyte response to tetanus toxoid among Indonesian men immunized with tetanus-diphtheria during extended chloroquine or primaquine prophylaxis. Journal of Infectious Diseases. 1997;176(6):1644-8.

22. Galan LEB, Santos NMD, Asato MS, Araújo JV, de Lima Moreira A, Araújo AMM, et al. Phase 2 randomized study on chloroquine, hydroxychloroquine or ivermectin in hospitalized patients with severe manifestations of SARS-CoV-2 infection. Pathog Glob Health. 2021:1-8.

23. Giannini EH, Brewer EJ, Kuzmina N, Alekseev L, Shokh BP. Characteristics of responders and nonresponders to slow-acting antirheumatic drugs in juvenile rheumatoid arthritis. Arthritis and Rheumatism. 1988;31(1):15-20.

24. Graber TG, Borack MS, Reidy PT, Volpi E, Rasmussen BB. Essential amino acid ingestion alters expression of genes associated with amino acid sensing, transport, and mTORC1 regulation in human skeletal muscle. Nutrition & metabolism. 2017;14(1):1-11.

25. Hugosson E, Tarimo D, Troye-Blomberg M, Montgomery SM, Premji Z, BJÖRKMAN A. Antipyretic, parasitologic, and immunologic effects of combining sulfadoxine/pyrimethamine with chloroquine or paracetamol for treating uncomplicated Plasmodium falciparum malaria. The American journal of tropical medicine and hygiene. 2003;69(4):366-71.

26. Ibrahim AM, Elhag E, Mustafa S. Ketotifen in treatment of uncomplicated falciparum malaria. Saudi medical journal. 2000;21(3):257-65.

27. Johansson E, Forsberg K, Johnsson H. Clinical and experimental evaluation of the thromboprophylactic effect of hydroxychloroquine sulfate after total hip replacement. Pathophysiology of Haemostasis and Thrombosis. 1981;10(2):89-96.

28. Kerckhove CV, Giannini EH, Lovell DJ. Temporal patterns of response to d‐penicillamine, hydroxychloroquine, and placebo in juvenile rheumatoid arthritis patients. Arthritis & Rheumatism. 1988;31(10):1252-8.

29. Kim HA, Choi HJ, Baek HJ, Lim MJ, Park W, Lee J, et al. Nonsteroidal antiinflammatory drugs (NSAID) versus NSAID with hydroxychloroquine in treatment of chemotherapy-related arthropathy: open-label multicenter pilot study. J Rheumatol. 2012;39(9):1902-3.

30. Kollaritsch H, Que JU, Kunz C, Wiedermann G, Herzog C, Cryz Jr SJ. Safety and immunogenicity of live oral cholera and typhoid vaccines administered alone or in combination with antimalarial drugs, oral polio vaccine, or yellow fever vaccine. Journal of Infectious Diseases. 1997;175(4):871-5.

31. Lee LS, Leow MK, Xu Y, Wilder-Smith A, Cheung YB, Paton NI. Low-dose chloroquine is associated with favourable effects on lipoprotein metabolism without significant influence on insulin resistance. Diabetic Medicine. 2016;33(3):404-5.

32. Group* CHS. A randomized study of the effect of withdrawing hydroxychloroquine sulfate in systemic lupus erythematosus. New England Journal of Medicine. 1991;324(3):150-4.

33. Leroux-Roels G, Bourguignon P, Willekens J, Janssens M, Clement F, Didierlaurent AM, et al. Immunogenicity and safety of a booster dose of an investigational adjuvanted polyprotein HIV-1 vaccine in healthy adults and effect of administration of chloroquine. Clinical and Vaccine Immunology. 2014;21(3):302-11.

34. Lewis AN, Ponnampalam JT. Suppression of malaria with monthly administration of combined sulphadoxine and pyrimethamine. Ann Trop Med Parasitol. 1975;69(1):1-12.

35. Li R, Zhao J-X, Su Y, He J, Chen L-N, Gu F, et al. High remission and low relapse with prolonged intensive DMARD therapy in rheumatoid arthritis (PRINT): a multicenter randomized clinical trial. Medicine. 2016;95(28).

36. Liu E, Liu Z, Zhou Y. Feasibility of hydroxychloroquine adjuvant therapy in pregnant women with systemic lupus erythematosus. Biomedical Research (India). 2018;29:980-3.

37. Marko JL, Chourishi A, Pandey SP. Comparison of efficacy of chloroquine alone, azithromycin alone, and chloroquine azithromycin combination for the treatment of uncomplicated Plasmodium Falciparum Malaria. International Journal of Pharmaceutical Sciences Review and Research. 2011;11(1):7-12.

38. Meng J. Long-term effects of of hydroxychloroquine on metabolism of serum lipids and left ventricular structure and fuction in patients of systemic lupus erythematosus. Lupus Science and Medicine. 2017;4:A120-A1.

39. Mottaghi P, Karimzade H. Does chloroquine decrease liver enzyme abnormalities induced by methoterexate in patients with rheumatoid arthritis? Journal of Research in Medical Sciences. 2005;10(3):135-8.

40. Mullick S, Das S, Guha SK, Bera DK, Sengupta S, Roy D, et al. Efficacy of chloroquine and sulphadoxine-pyrimethamine either alone or in combination before introduction of ACT as first-line therapy in uncomplicated Plasmodium falciparum malaria in Jalpaiguri District, West Bengal, India. Trop Med Int Health. 2011;16(8):929-35.

41. Murray SM, Down CM, Boulware DR, Stauffer WM, Cavert WP, Schacker TW, et al. Reduction of immune activation with chloroquine therapy during chronic HIV infection. J Virol. 2010;84(22):12082-6.

42. Naarding M, Luchters S, Vyankandondera J, Wit F, Veldhuijzen N, Kankindi B, et al. Use of chloroquine in reducing mother to child transmission of HIV-1 during breastfeeding. Retrovirology. 2009;6.

43. Ndyomugyenyi R, Magnussen P. Chloroquine prophylaxis, iron/folic-acid supplementation or case management of malaria attacks in primigravidae in western Uganda: effects on congenital malaria and infant haemoglobin concentrations. Ann Trop Med Parasitol. 2000;94(8):759-68; discussion 69-70.

44. Onyeji CO, Toriola TA, Ogunbona FA. Lack of pharmacokinetic interaction between chloroquine and imipramine. Ther Drug Monit. 1993;15(1):43-6.

45. Padmakumar B, Jayan J, Menon R, Krishnankutty B, Payippallil R, Nisha R. Comparative evaluation of four therapeutic regimes in Chikungunya arthritis: A prospective randomized parallel-group study. Indian Journal of Rheumatology. 2009;4:94-101.

46. Paxton WA, Naarding MA, Wit FWW, Veldhuijzen NJ, Chersich MF, Kankindi BK, et al. Chloroquine Administration in Breastfeeding Mothers Associates with Increased HIV-1 Plasma Viral Loads. medRxiv. 2020:2020.04.29.20085308.

47. Pinto AYdN, Azevedo CH, Silva JBd, Souza JMd. Assessment of chloroquine single dose treatment of malaria due to Plasmodium vivax in Brazilian Amazon. Revista do Instituto de Medicina Tropical de São Paulo. 2003;45:327-31.

48. Powrie JK, Smith GD, Shojaee-Moradie F, Sönksen PH, Jones RH. Mode of action of chloroquine in patients with non-insulin-dependent diabetes mellitus. Am J Physiol. 1991;260(6 Pt 1):E897-904.

49. Pukrittayakamee S, Tarning J, Jittamala P, Charunwatthana P, Lawpoolsri S, Lee SJ, et al. Pharmacokinetic interactions between primaquine and chloroquine. Antimicrob Agents Chemother. 2014;58(6):3354-9.

50. Salah MT, Mohammed MM, Himeidan YE, Malik EM, Elbashir MI, Adam I. A randomized comparison of sulphadoxine-pyrimethamine and combination of sulphadoxine pyrimethamine with chloroquine in the treatment of uncomplicated falciparum malaria in Eastern Sudan. Saudi Med J. 2005;26(1):147-8.

51. Sheikhbahaie F, Amini M, Gharipour M, Aminoroaya A, Taheri N. The effect of hydroxychloroquine on glucose control and insulin resistance in the prediabetes condition. Adv Biomed Res. 2016;5:145.

52. Solomon DH, Garg R, Lu B, Todd DJ, Mercer E, Norton T, et al. Effect of hydroxychloroquine on insulin sensitivity and lipid parameters in rheumatoid arthritis patients without diabetes mellitus: A randomized, blinded crossover trial. Arthritis Care and Research. 2014;66(8):1246-51.

53. Somer M, Kallio J, Pesonen U, Pyykkö K, Huupponen R, Scheinin M. Influence of hydroxychloroquine on the bioavailability of oral metoprolol. Br J Clin Pharmacol. 2000;49(6):549-54.

54. Talisuna AO, Nalunkuma-Kazibwe A, Bakyaita N, Langi P, Mutabingwa TK, Watkins WW, et al. Efficacy of sulphadoxine-pyrimethamine alone or combined with amodiaquine or chloroquine for the treatment of uncomplicated falciparum malaria in Ugandan children. Trop Med Int Health. 2004;9(2):222-9.

55. Tarimo DS, Minjas JN, Bygbjerg IC. Sulfadoxine-pyrimethamine monotherapy in Tanzanian children gives rapid parasite clearance but slow fever clearance that is improved by chloroquine in combination therapy. Trop Med Int Health. 2002;7(7):592-8.

56. Taylor WR, Widjaja H, Richie TL, Basri H, Ohrt C, Tjitra, et al. Chloroquine/doxycycline combination versus chloroquine alone, and doxycycline alone for the treatment of Plasmodium falciparum and Plasmodium vivax malaria in northeastern Irian Jaya, Indonesia. Am J Trop Med Hyg. 2001;64(5-6):223-8.

57. Tsakonas E, Joseph L, Esdaile JM, Choquette D, Senécal JL, Cividino A, et al. A long-term study of hydroxychloroquine withdrawal on exacerbations in systemic lupus erythematosus. The Canadian Hydroxychloroquine Study Group. Lupus. 1998;7(2):80-5.

58. Updike SJ, Eichman PL. Infectious mononucleosis treated with chloroquine. A double-blind study of 40 cases. Am J Med Sci. 1967;254(1):69-70.

59. Wetsteyn JC, de Geus A. Comparison of three regimens for malaria prophylaxis in travellers to east, central, and southern Africa. Bmj. 1993;307(6911):1041-3.

60. Pan H, Peto R, Henao-Restrepo AM, Preziosi MP, Sathiyamoorthy V, Abdool Karim Q, et al. Repurposed Antiviral Drugs for Covid-19 - Interim WHO Solidarity Trial Results. N Engl J Med. 2021;384(6):497-511.

1. **References of ongoing studies**

1. Laurens MB, Mungwira RG, Nyirenda OM, Divala TH, Kanjala M, Muwalo F, et al. TSCQ study: a randomized, controlled, open-label trial of daily trimethoprim-sulfamethoxazole or weekly chloroquine among adults on antiretroviral therapy in Malawi: study protocol for a randomized controlled trial. Trials. 2016;17(1):322.

2. Mekinian A, Vicaut E, Cohen J, Bornes M, Kayem G, Fain O. [Hydroxychloroquine to obtain pregnancy without adverse obstetrical events in primary antiphospholipid syndrome: French phase II multicenter randomized trial, HYDROSAPL]. Gynecol Obstet Fertil Senol. 2018;46(7-8):598-604.

3. Olsen NJ, James JA, Arriens C, Ishimori ML, Wallace DJ, Kamen DL, et al. Study of Anti-Malarials in Incomplete Lupus Erythematosus (SMILE): study protocol for a randomized controlled trial. Trials. 2018;19(1):694.

4. Schreiber K, Breen K, Cohen H, Jacobsen S, Middeldorp S, Pavord S, et al. HYdroxychloroquine to Improve Pregnancy Outcome in Women with AnTIphospholipid Antibodies (HYPATIA) Protocol: A Multinational Randomized Controlled Trial of Hydroxychloroquine versus Placebo in Addition to Standard Treatment in Pregnant Women with Antiphospholipid Syndrome or Antibodies. Semin Thromb Hemost. 2017;43(6):562-71.

5. Griese M, Köhler M, Witt S, Sebah D, Kappler M, Wetzke M, et al. Prospective evaluation of hydroxychloroquine in pediatric interstitial lung diseases: Study protocol for an investigator-initiated, randomized controlled, parallel-group clinical trial. Trials. 2020;21(1):307.

6. Detert J, Klaus P, Listing J, Höhne-Zimmer V, Braun T, Wassenberg S, et al. Hydroxychloroquine in patients with inflammatory and erosive osteoarthritis of the hands (OA TREAT): study protocol for a randomized controlled trial. Trials. 2014;15(1):412.

7. Hartman O, Kovanen PT, Lehtonen J, Eklund KK, Sinisalo J. Hydroxychloroquine for the prevention of recurrent cardiovascular events in myocardial infarction patients: rationale and design of the OXI trial. Eur Heart J Cardiovasc Pharmacother. 2017;3(2):92-7.

8. Nct, Semenkovich FC. Metabolic Effects of Hydroxychloroquine. <https://clinicaltrialsgov/show/NCT02026232>. 2013(5).

9. Nct, Deane K, Holers M, Striebich C. Strategy to Prevent the Onset of Clinically-Apparent Rheumatoid Arthritis. <https://clinicaltrialsgov/show/NCT02603146>. 2015(5).

10. Euctr FR. Etude de la réduction des poussées de lupus érythémateux systémique par adaptation de la posologie de l'hydroxychloroquine à sa concentration sanguine. Etude randomisée multicentrique nationale. - PLUS. <http://wwwwhoint/trialsearch/Trial2aspx?TrialID=EUCTR2006-003025-93-FR>. 2007.

11. Nct. Efficacy Study of Hydroxychloroquine to Treat High-risk Coronary Artery Disease. <https://clinicaltrialsgov/show/NCT02874287>. 2016.

12. Pasquier E, de Saint-Martin L, Marhic G, Chauleur C, Bohec C, Bretelle F, et al. Hydroxychloroquine for prevention of recurrent miscarriage: study protocol for a multicentre randomised placebo-controlled trial BBQ study. BMJ Open. 2019;9(3):e025649.

13. Nct. Effect of Hydroxychloroquine on Atrial Fibrillation Recurrence. <https://clinicaltrialsgov/show/NCT03592823>. 2018.

14. Nct. Hydroxychloroquine in Individuals At-risk for Type 1 Diabetes Mellitus. <https://clinicaltrialsgov/show/NCT03428945>. 2018.

15. Nct. Hydroxychloroquine Versus Placebo: impact on Thrombotic Relapse in Primary Antiphospholipid Syndrome. <https://clinicaltrialsgov/show/NCT03540810>. 2018.

16. Nct. HCQ for the CVD in CKD. <https://clinicaltrialsgov/show/NCT03636152>. 2018.

17. Nct. Effect of Hydroxychloroquine Sulphate in Healthy Lean Females. <https://clinicaltrialsgov/show/NCT04005768>. 2019.

18. Nct. Hydroxychloroquine and Unexplained Recurrent Miscarriage. <https://clinicaltrialsgov/show/NCT04228263>. 2020.

19. Nct. HYDROxychloroquine in Syndrome Primary AntiPhospholipid. <https://clinicaltrialsgov/show/NCT04275778>. 2020.

20. ChiCtr. A randomized controlled trial for hydroxychloroquine sulfate in the treatment of idiopathic membranous nephropathy. <http://wwwwhoint/trialsearch/Trial2aspx?TrialID=ChiCTR1900021757>. 2019.

21. Euctr DK. Plaquenil treatment of recurrent precnancy losses – a randomized, placebo controlled study. <http://wwwwhoint/trialsearch/Trial2aspx?TrialID=EUCTR2016-004981-24-DK>. 2017.

22. Euctr FI. Hydroxychloroquine in the prevention of cardiovascular events in high risk patients. <http://wwwwhoint/trialsearch/Trial2aspx?TrialID=EUCTR2015-000233-73-FI>. 2015.

23. Euctr FR. Essai prospectif Randomisé contre placebo en double aveugle de l’hydroxychloroQUine dans le traitement du syndrome de sJOgren primitif (JOQUER) - JOQUER. <http://wwwwhoint/trialsearch/Trial2aspx?TrialID=EUCTR2007-005218-38-FR>. 2007.

24. Nct. Chloroquine Prevention of Coronavirus Disease (COVID-19) in the Healthcare Setting. <https://clinicaltrialsgov/show/NCT04303507>. 2020.

25. Euctr NL. Leflunomide and Hydroxychloroquine combination therapy for primary Sjogren's Syndrome. <http://wwwwhoint/trialsearch/Trial2aspx?TrialID=EUCTR2014-003140-12-NL>. 2015.

1. **References of studies we could not have full access.**

1. Mutabingwa TK, Malle LN, de Geus A, Oosting J. Malaria chemosuppression in pregnancy. I. The effect of chemosuppressive drugs on maternal parasitaemia. Trop Geogr Med. 1993;45(1):6-14.

2. Mutabingwa TK, Malle LN, Eling WM, Verhave JP, Meuwissen JH, de Geus A. Malaria chemosuppression in pregnancy. III. Its effects on the maternal malaria immunity. Trop Geogr Med. 1993;45(3):103-9.

3. Chen FY, Xue B, Wang H. [Analysis of the clinical efficacy of yiqi fumai injection combined hydroxychloroquine sulfate tablet for treating Sjogren's syndrome]. Zhongguo Zhong Xi Yi Jie He Za Zhi. 2012;32(12):1621-3.

4. Hansen EH, Jessing P, Lindewald H. Hydroxychloroquine sulphate in prevention of deep venous thrombosis following fracture of the hip, pelvis, or thoracolumbar spine. Journal of Bone and Joint Surgery - Series A. 1976;58(8):1089-93.

5. Ogunwande SA. Malarial chemoprophylaxis and the healing of periodontal lesions. Clin Prev Dent. 1991;13(2):5-8.

6. Ogwang S, Engl M, Vigl M, Kollaritsch H, Wiedermann G, Wernsdorfer WH. Clinical and parasitological response of Plasmodium falciparum to chloroquine and sulfadoxine/pyrimethamine in rural Uganda. Wiener Klinische Wochenschrift, Supplement. 2003;115(3):45-9.

7. Williams HJ, Egger MJ, Singer JZ, Willkens RF, Kalunian KC, Clegg DO, et al. Comparison of hydroxychloroquine and placebo in the treatment of the arthropathy of mild systemic lupus erythematosus. Journal of Rheumatology. 1994;21(8):1457-62.

8. Wolde B, Pickering J, Wotton K. Chloroquine chemoprophylaxis in children during peak transmission period in Ethiopia. Journal of Tropical Medicine and Hygiene. 1994;97(4):215-

9. Barry M, Patterson JE, Tirrell S, Cullen MR, Shope RE. The effect of chloroquine prophylaxis on yellow fever vaccine antibody response: Comparison of plaque reduction neutralization test and enzyme-linked immunosorbent assay. American Journal of Tropical Medicine and Hygiene. 1991;44(1):79-82.

10. Chrisman OD, Snook GA, Wilson TC, Short JY. Prevention of venous thromboembolism by administration of hydroxychloroquine. A preliminary report. Journal of Bone and Joint Surgery - Series A. 1976;58(7):918-20.

11. Clegg DO, Dietz F, Duffy J, Willkens RF, Hurd E, Germain BF, et al. Safety and efficacy of hydroxychloroquine as maintenance therapy for rheumatoid arthritis after combination therapy with methotrexate and hydroxychloroquine. Journal of Rheumatology. 1997;24(10):1896-902.

12. Cooke ED, Dawson MHO, Ibbotson RM. Failure of orally administered hydroxychloroquine sulphate to prevent venous thromboembolism following elective hip operations. Journal of Bone and Joint Surgery - Series A. 1977;59(4):496-500.

13. Gyhrs A, Pedersen BK, Bygbjerg I, Henrichsen J, Heron I, Petersen I, et al. The effect of prophylaxis with chloroquine and proguanil on delayed-type hypersensitivity and antibody production following vaccination with diphtheria, tetanus, polio, and pneumococcal vaccines. American Journal of Tropical Medicine and Hygiene. 1991;45(5):613-8.

14. Porter DR, Capell HA, Hunter J. Combination therapy in rheumatoid arthritis - No benefit of addition of hydroxychloroquine to patients with a suboptimal response to intramuscular gold therapy. Journal of Rheumatology. 1993;20(4):645-9.

15. Wu TK, Tsapogas MJ, Jordan FR. Prophylaxis of deep venous thrombosis by hydroxychloroquine sulfate and heparin. Surg Gynecol Obstet. 1977;145(5):714-8.

16. Tsapogas MJ, Wu KT, Jordan FR. Controlled study on hydrochloroquine sulfate and heparin in the prophylaxis of deep venous thrombosis1976. No.362 p.

1. **Search Strategies - 11/April/2021**

**PubMed**

#1 "Hydroxychloroquine"[Mesh] OR (Oxychlorochin) OR (Oxychloroquine) OR (Hydroxychlorochin) OR (Plaquenil) OR (Hydroxychloroquine Sulfate) OR (Hydroxychloroquine Sulfate (1:1) Salt)

# 2"Chloroquine"[Mesh] OR (Chlorochin) OR (Chingamin) OR (Khingamin) OR (Nivaquine) OR (Chloroquine Sulfate) OR (Sulfate, Chloroquine) OR (Chloroquine Sulphate) OR (Sulphate, Chloroquine) OR (Aralen) OR (Arequin OR (Arequin) OR (Arechine)

#3 "chloroquine diphosphate" [Supplementary Concept] OR (chingamin phosphate) OR (unspecified phosphate of chloroquine diphosphate) OR (delagil) OR (khingamin phosphate) OR (arechin) OR (chloroquine phosphate) OR (Resochin) OR (chloroquine diphosphate, (+-)-isomer) OR (chloroquine diphosphate, (-)-isomer) OR (chloroquine bis(dihydrogenphosphate) dihydrate) OR (chloroquine diphosphate, (+)-isomer)

# *4 **randomized controlled trial[pt] OR controlled clinical trial[pt] OR randomized[tiab] OR placebo[tiab] OR clinical trials as topic[mesh:noexp] OR randomly[tiab] OR trial[ti] NOT (animals[mh] NOT humans [mh]))**

***sensitivity- and precision-maximizing version (2008 revision); PubMed format – Cochrane**

**(("chloroquine diphosphate" [Supplementary Concept] OR (chingamin phosphate) OR (unspecified phosphate of chloroquine diphosphate) OR (delagil) OR (khingamin phosphate) OR (arechin) OR (chloroquine phosphate) OR (Resochin) OR (chloroquine diphosphate, (+-)-isomer) OR (chloroquine diphosphate, (-)-isomer) OR (chloroquine bis(dihydrogenphosphate) dihydrate) OR (chloroquine diphosphate, (+)-isomer)) OR ("Chloroquine"[Mesh] OR (Chlorochin) OR (Chingamin) OR (Khingamin) OR (Nivaquine) OR (Chloroquine Sulfate) OR (Sulfate, Chloroquine) OR (Chloroquine Sulphate) OR (Sulphate, Chloroquine) OR (Aralen) OR (Arequin OR (Arequin) OR (Arechine))) OR ("Hydroxychloroquine"[Mesh] OR (Oxychlorochin) OR (Oxychloroquine) OR (Hydroxychlorochin) OR (Plaquenil) OR (Hydroxychloroquine Sulfate) OR (Hydroxychloroquine Sulfate (1:1) Salt))) AND ((randomized controlled trial[pt] OR controlled clinical trial[pt] OR randomized[tiab] OR placebo[tiab] OR clinical trials as topic[mesh:noexp] OR randomly[tiab] OR trial[ti] NOT (animals[mh] NOT humans [mh])))**

**Total= 1543**

**EMBASE**

'hydroxychloroquine'/exp OR '7 chloro 4 [4 [ethyl (2 hydroxyethyl) amino] 1 methylbutylamino] quinoline' OR '7 chloro 4 [4 [ethyl (2 hydroxyethyl) amino] 1 methylbutylamino] quinoline diphosphate' OR 'chloroquinol' OR 'ercoquin' OR 'hydrochloroquine' OR 'hydrocloroquine' OR 'oxychloroquine' OR 'quensyl' OR 'sn 8137'

'hydroxychloroquine sulfate'/exp OR '1 (7 chloro 4 quinolylamino) 3 diethylamino 2 propanol sulfate' OR '1 (7 chloro 4 quinolylamino) 3 diethylamino 2 propanol sulphate’ OR '2 [ [4 [ (7 chloro 4 quinolinyl) amino] pentyl] (ethyl) amino] ethanol sulfate' OR '2 [ [4 [ (7 chloro 4 quinolinyl) amino] pentyl] (ethyl) amino] ethanol sulphate' OR '2 [ [4 [ (7 chloroquinolin 4 yl) amino] pentyl] (ethyl) amino] ethanol sulfate' OR '2 [ [4 [ (7 chloroquinolin 4 yl) amino] pentyl] (ethyl) amino] ethanol sulphate' OR '7 chloro 4 (3 diethylamino 2 hydroxypropylamino) quinoline sulfate' OR '7 chloro 4 (3 diethylamino 2 hydroxypropylamino) quinoline sulphate' OR 'dimard' OR 'erquin' OR 'evoquin' OR 'geniquin' OR 'hydroxychloroquine sulphate' OR 'oxiklorin' OR 'oxychloroquine sulfate' OR 'oxychloroquine sulphate' OR 'plaquenil' OR 'plaquenil sulfate' OR 'plaquenil sulphate' OR 'plaquinol' OR 'toremonil' OR 'yuma'

'chloroquine'/exp OR '4 (4 diethylamino 1 methylbutylamino) 7 chlorchinolin diphosphate' OR '4 (4 diethylamino 1 methylbutylamino) 7 chlorchinolin sulfate' OR '4 (4 diethylamino 1 methylbutylamino) 7 chlorchinolin sulphate' OR '4 (4 diethylamino 1 methylbutylamino) 7 chloroquinoline’ OR ‘7 chloro 4 (4 diethylamino 1 methylbutylamino) quinoline’ OR ‘7 chloro 4 (4 diethylamino 1 methylbutylamino) quinoline diphosphate’ OR '7 chloro 4 (4 diethylamino 1 methylbutylamino) quinoline' OR 'a-cq' OR 'amokin' OR 'amokine' OR 'anoclor' OR 'aralan' OR 'aralen' OR 'aralen hydrochloride’ OR ‘aralen phosphate’ OR ‘aralene’ OR ‘arechin’ OR ‘arechine’ OR ‘arequine’ OR ‘arthrochin’ OR ‘arthrochine’ OR ‘arthroquine’ OR ‘artrichin’ OR ‘artrichine’ OR ‘artriquine’ OR ‘avloclor’ OR ‘avoclor’ OR ‘bemaphata’ OR ‘bemaphate’ OR ‘bemasulph’ OR ‘bipiquin’ OR 'cadiquin’ OR 'chemochin' OR 'chemochine' OR 'chingamine' OR 'chingaminum' OR 'chloraquine' OR 'chlorochi' OR 'chlorochine' OR 'chlorofoz' OR 'chloroquin' OR 'chloroquin phosphate' OR 'chloroquine diphosphate' OR 'chloroquine disulfate' OR 'chloroquine disulphate' OR 'chloroquine hydrochloride' OR 'chloroquine phosphate' OR 'chloroquine streuli' OR 'chloroquine sulfate' OR 'chloroquine sulphate’ OR 'chloroquinesulphate' OR 'chloroquini diphosphas’ OR 'chloroquinum diphosphoricum' OR 'chlorquin' OR 'chlorquine' OR 'choloquine' OR 'choroquine sulfate' OR ‘choroquine sulphate’ OR 'cidanchin’ OR ‘clo-kit junior' OR 'clorichina' OR 'clorichine' OR 'cloriquine' OR 'clorochina' OR 'delagil' OR 'delagyl' OR 'dichinalex' OR 'diclokin' OR 'diquinalex' OR 'diroquine' OR 'emquin' OR 'genocin' OR 'gontochin' OR 'gontochine' OR 'gontoquine' OR 'heliopar' OR 'imagon' OR 'iroquine' OR 'klorokin' OR 'klorokine' OR 'klorokinfosfat' OR 'lagaquin' OR 'malaquin' OR 'malarex' OR 'malarivon' OR 'malaviron' OR 'maliaquine' OR 'maquine' OR 'mesylith' OR 'mexaquin' OR 'mirquin’ OR 'nivachine' OR 'nivaquin' OR 'nivaquine' OR 'nivaquine (b)' OR 'nivaquine b' OR 'nivaquine dp' OR 'nivaquine forte' OR ‘p roquine' OR 'quinachlor' OR 'quingamine' OR 'repal' OR 'resochen' OR 'resochene' OR 'resochin' OR 'resochin junior' OR 'resochina' OR 'resochine' OR 'resochinon' OR 'resoquina' OR 'resoquine' OR 'reumachlor' OR 'roquine' OR 'rp 3377' OR 'rp3377' OR 'sanoquin' OR 'sanoquine' OR 'silbesan' OR 'siragan' OR 'sirajan' OR 'sn 7618' OR 'sn7618' OR 'solprina' OR 'solprine' OR 'tresochin' OR 'tresochine' OR 'tresoquine' OR 'trochin' OR 'trochine' OR 'troquine' OR '7618' OR 'w7618' OR 'win 244' OR 'win244'

Filter

best optimization of sensitivity and specificity*

random*:ab,ti OR placebo*:de,ab,ti OR (double NEXT/1 blind*):ab,ti

*Wong SS, Wilczynski NL, Haynes RB. Developing optimal search strategies for detecting clinically sound treatment studies in EMBASE. Journal of the Medical Library Association : JMLA. 2006 Jan;94(1):41-7

Total=3726

**CENTRAL**

Search Name:

Date Run: 20/04/2020 10:37:49

Comment:

ID Search Hits

#1 MeSH descriptor: [Hydroxychloroquine] explode all trees 302

#2 'Hydroxychloroquine Sulfate Salt' OR 'Hydroxychloroquine Sulfate' OR 'Plaquenil' OR 'Hydroxychlorochin' OR 'Oxychlorochin' OR 'Oxychloroquine' 104

#3 MeSH descriptor: [Chloroquine] explode all trees 976

#4 'Chloroquine Sulphate' OR 'Chloroquine Sulfate' OR 'Sulphate, Chloroquine' OR 'Sulfate, Chloroquine' 35

#5 #1 OR #2 OR #3 OR #4 1055

**BVS (Lilacs, IBECS, Hanseniase, Sec Muni SP, Coleciona Sus)**

#1 MH:"**Hidroxicloroquina**" OR (Oxicloroquina) OR MH: D03.633.100.810.050.180.350$

#2 MH:"**Cloroquina**" **OR MH:** D03.633.100.810.050.180$

#1 OR #2= 156

**Trip Database**

Hydroxychloroquine, filter controlled trial 199

Chloroquine, filter controlled trial 549

**SCOPUS**

LL ( " Hydroxychloroquine" )  OR  ALL ( " Chloroquine " )  AND  ALL ( "randomized controlled trial" )  AND NOT  INDEX ( medline )  AND  ( LIMIT-TO ( DOCTYPE ,  "ar" ) )

Total= 866
